# The low frequency pressure pulsation and control of the open-jet wind tunnel

**DOI:** 10.1038/s41598-022-22080-9

**Published:** 2022-11-09

**Authors:** Xingjun Hu, Yufei Luo, Jiu Leng, Peng Guo, Tianming Yu, Jingyu Wang

**Affiliations:** State Key Laboratory of Automotive Simulation and Control, Jilin University, Changchun, 130022 China

**Keywords:** Engineering, Mechanical engineering

## Abstract

An open jet wind tunnel has low-frequency pressure pulsation in common wind speed range due to its unique structural form, which seriously damages the quality of flow field in the test section. The low-frequency pressure fluctuation performance and control mechanism of Jilin University open jet and return flow wind tunnel are investigated by experiments and numerical simulation. The results show that the low-frequency pressure fluctuation is a narrow pulse phenomenon that only occurs in certain intervals, and several velocity intervals may be found in the same wind tunnel. The reliability of the numerical simulation is verified by comparing the peak frequency and amplitude of pressure fluctuation in numerical simulation and wind tunnel tests. A simplified model similar to and amplifying the phenomenon is established. The flow structure and vortex evolution are analyzed via detached eddy simulation. In the test section, large-scale shedding vortices are formed at the nozzle exit, introducing periodic pulsating instantaneous velocity and acting with the collector to form an edge-feedback. This acoustic feedback forms resonance with the pipeline circuit, resulting in poor flow field quality. In accordance with the mechanism of nozzle jet, two methods of controlling pulsation are proposed: spoiler and flow-follow device. The study shows that the effects of two methods are abrupt, and the frequency of pressure pulsation is changed. The spoiler destroys the complete structure of vortex ring in free jet and develops into a complementary double vortex ring structure, which is highly sensitive to size factors. The flow-follow device supplements the velocity loss of the free jet at the nozzle and develops into a double vortex ring with master–slave structure in the middle of the test section. Its vibration reduction effect is greatly affected by the flow velocity. It takes effect in an appropriate range where the flow velocity is higher than the nozzle velocity. If the follow velocity is extremely low, the flow-follow device cannot change the original jet structure. If the follow velocity is extremely high, the momentum of the fan will be greatly reduced, the flow field will be unstable, and another order of pulsation may be induced. This work lays a solid foundation for further understanding the aerodynamic characteristics and optimization mechanism of open jet wind tunnel.

## Introduction

As an important means of aerodynamic test, the wind tunnel plays a vital role in the development and design process of aerospace, ground transportation, large-scale bridge construction, and other fields^1^. The objective of this study is the three-fourth open jet return flow wind tunnel, where theflow field quality is free from the disturbance of the external atmospheric environment. It has the characteristics of airflow kinetic energy circulation and low energy consumption in operation^2–4^.

However, the structure of the open jet and return flow wind tunnel determines that the airflow from the nozzle will form an unstable free jet structure. As an excitation, it matches with the acoustic mode of the wind tunnel loop, resulting in resonance phenomenon, which leads to strong periodic pulsations in the approximately steady flow field. This phenomenon is called low-frequency pressure pulsation because its frequency is lower than 20 Hz^5^. The fluctuation of infrasound wave in the wind tunnel will destroy the space–time uniformity of the core area field, threaten the safety of the tunnel structure, and affect the accuracy of the experimental results^6,7^.

In the twentieth century, Jacobs E.N. found that pressure pulsation occurs in a certain range of wind speed in the test of open wind tunnel and considered that this phenomenon is related to the size of wind tunnel, nozzle structure, and collector structure^8^. Scholars believe that the pulsation of the whole loop is different from the simple sound pressure radiation. This condition is a macro convection phenomenon, where the pressure is related to the velocity^9^, and this phenomenon is caused by the unstable structure inside the jet shear layer^10^. Subsequently, the American Academy of Aeronautics and Astronautics focused on the interaction between the nozzle and the collector, and named it edge tone-type feedback^11^. The National Aeronautics and Space Administration added different forms of additional structures at the nozzle of open jet wind tunnel. The test shows that the device can destroy the original vortex structure of the jet shear layer and greatly alleviate the pressure pulsation phenomenon^12^. DNW wind tunnel has conducted similar research on the nozzle and found that its installation quantity and size will affect the cushioning^13^. In 2014, Blumrich et al. modified the nozzle attachment of the FKFS acoustical wind tunnel to reduce pressure fluctuations while reducing turbulent noise^14^. Jia Q studied the pressure balance port and the collector baffles at different angles in a parking chamber based on wind tunnel tests and computational fluid dynamics (CFD). He believed that the reasonable position of pressure balance port and the angle of the collector are beneficial to reduce the pressure pulsation phenomenon^15^.

Most flow phenomena can be described by the continuity equation (i.e., mass conservation equation) and Navier–Stokes (N–S) equation (i.e., momentum conservation equation). Three CFD methods are mainly used to solve N–S equations: direct numerical simulation (DNS), large eddy simulation (LES), and Reynolds averaged NS simulation (RANS)^16^. Although DNS has the highest accuracy, it takes up a large amount of physical memory and uses considerable high-performance processors to calculate complex flow field for a long time. Thus, only simple mechanism study is conducted with it^17^. The indirect numerical simulation method simplifies the turbulent structure at the beginning of calculation and then solves the three equations. Compared with the original equation, a new additional Reynolds stress term appears in the N–S equation of Reynolds averaging method. Other turbulence models, including Reynolds stress model and eddy viscosity model, need to be introduced to make the equation closed. This method is widely used in engineering due to its fast calculation time and low resource consumption. Large eddy simulation (LES) is between DNS and RANS. It uses a filtering function to separate vortices into large scale and small scale. For the former, DNS is used to calculate the N–S equation directly. For the latter, a subgrid scale model is used to simulate the effect of small vortices on large vortices. The calculation accuracy and quantity are between DNS and RANS.

With the development of CFD method, an increasing number of scholars apply numerical methods to optimize and evaluate wind tunnel components^18–20^. Joel^21^ conducted a numerical simulation on flow field quality, background noise, and structure optimization of Chrysler 3:8 pilot model wind tunnel, selected reasonable contraction ratio, improved the velocity distribution uniformity of nozzle section, and reduced the thickness of inflow boundary layer^22–24^. John et al.^25,26^ investigated the corner deflector and diffuser section with deflector in a wind tunnel circuit through simulation. The results show that these deflectors can effectively reduce the air separation in pipe and increase the flow field uniformity in the test section. Jia Q used LES to simulate the vortex structure in the jet shear layer at the nozzle when the low-frequency pressure fluctuation occurs, which is in good agreement with the particle image velocimetry test results^27^. The above simulation methods can provide some reference for the numerical simulation study of pulsation phenomenon in this study. This study uses the DES model based on shear stress transport k–ω model. The DES model is a hybrid model combining RANS and LES models. It uses LES to solve the flow outside the boundary layer and Reynolds averaging to solve the flow inside the boundary layer^28^. This model saves more computing resources than LES, and the flow field information is more complete, which conforms to the calculation accuracy of this study. Therefore, this method is used for transient calculation.

The above study is limited to retrofitted existing wind tunnels by tests. Few studies are relatively conducted on how to control the general phenomenon of low-frequency pressure fluctuation in open jet wind tunnels in the design stage, and the results are limited to the preliminary explanation of the fluctuation phenomenon. How to effectively avoid low-frequency pressure fluctuation and establish a good flow field quality in the full wind speed range are the main problems faced by an open jet and return flow wind tunnel. In this study, the mechanism of low-frequency pressure fluctuation is studied by 3D DES model of Jilin University opening wind tunnel. The accuracy of the numerical results is verified by comparing the peak frequency and amplitude of pressure fluctuation obtained from CFD and wind tunnel tests. A simplified wind tunnel model with universal characteristics and amplified amplitude is established based on the original model. The vibration mitigation mechanism of an open jet wind tunnel with spoilers and the flow-follow device is studied based on the generation mechanism of acoustic feedback. This work provides theoretical support for a deeper and more comprehensive understanding of low-frequency pressure fluctuation characteristics of open jet wind tunnel.

## Experiment and simulation

### Wind tunnel model parameters

**Figure 1:**
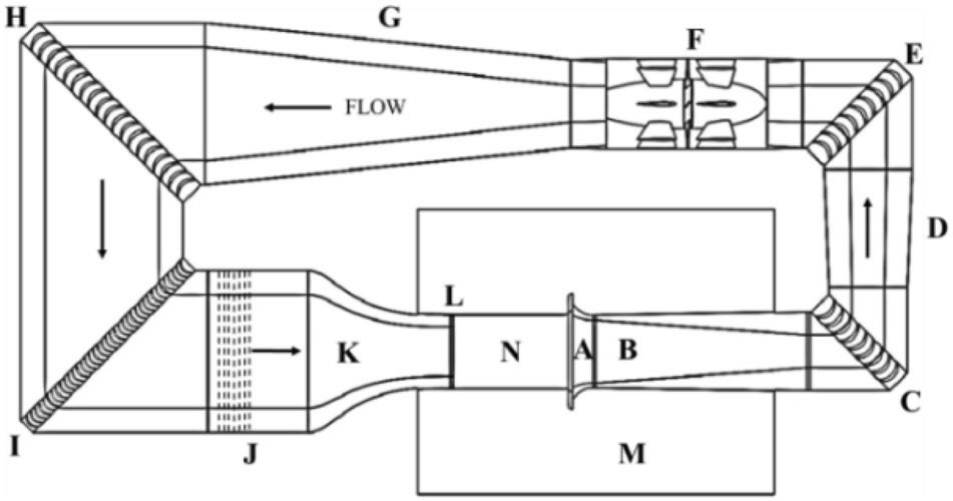
Top view of the wind tunnel in Jilin University general layout. The arrows indicate the direction of local flow. The key components are as follows: (A) Collector, (B) First diffuser, (C) First turning corner, (D) Second diffuser, (E) Second turning corner, (F) Axial fan, (G) Third diffuser, (H) Third turning corner, (I) Fourth turning corner, (J) Settling chamber, (K) Contraction, (L) Nozzle, (N) Test section, (M) Plenum chamber.

The automobile wind tunnel of Jilin University is a low-speed, open jet, and return flow wind tunnel. The overall layout is shown in Fig. 1. The wind tunnel is built in the testing hall. The tunnel structure is suspended on the ground by brackets. The control room is arranged on the side of the test section. The sound source propagation is blocked by glass and sound absorbing materials. Rectification devices, such as honeycomb net and damping net, are used. The design includes a test space of 8 m (length) * 4 m (width) * 2.2 m (height), and the maximum speed is 60 m/s to meet a wide range of application requirements. The length of the circuit axis is 114 m, the length of the long axis is 43 m, and the short axis is 14 m. The contraction ratio is 5.17, and the diffusion ratio is 1.84.

### Experimental setup

#### The testing equipment

**Figure 2:**
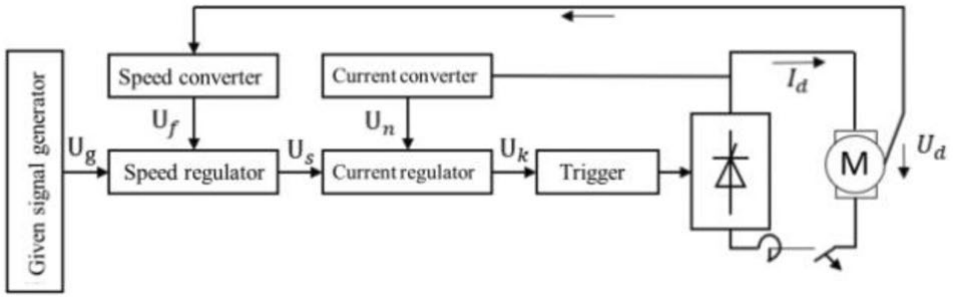
The dual closed loop Speed-controlled System.

The data acquisition of this test includes wind tunnel control and measurement system. The wind tunnel control and measurement system transmits commands through the host computer and the lower machine. The speed detected by the pitot tube at the nozzle is used as the target variable to drive the DC servo motor system to complete the closed-loop control. The control flow is shown in Fig. 2. The velocity at the nozzle is collected by NI pressure data acquisition system. Its accuracy is 0.010% fs. The sampling frequency of differential pressure sensor is 10 Hz and the sampling time is 20 s.

**Figure 3:**
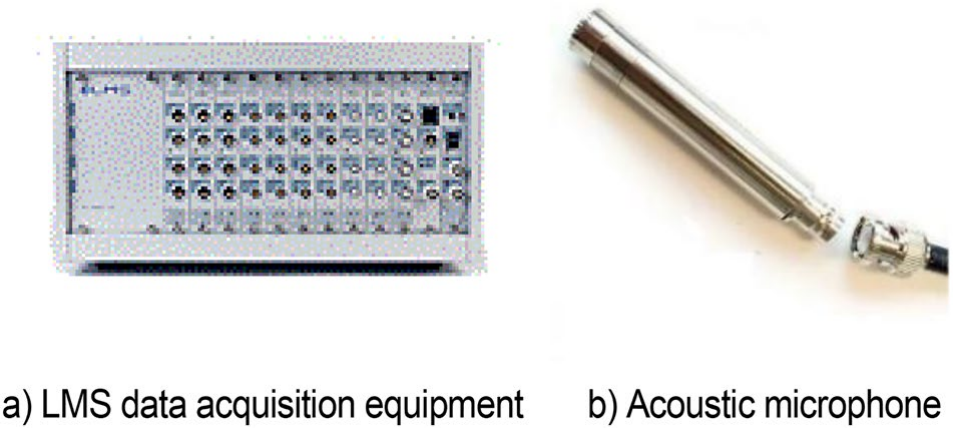
Acoustic acquisition device.

The acoustic measuring device mainly includes PCB 1/2-inch acoustic microphone, LMS SCADAS vibration and noise data acquisition system, BNC test connection line, and fixing bracket, as shown in Fig. 3. A single chassis of LMS SCADAS vibration can accommodate up to 120 channels. The maximum continuous transmission reaches 8 M sampling points/second. The sampling rate of each channel is as high as 204.8 kHz. The sampling channel is a single channel with differential wiring. The sampling frequency is 2048 Hz, and the sampling time is 10 s. Sampling is performed four times under the same working condition to ensure that the test is correct and no abnormal data are found. The average of four times is taken as the test result.

#### Distribution of monitoring points

**Figure 4:**
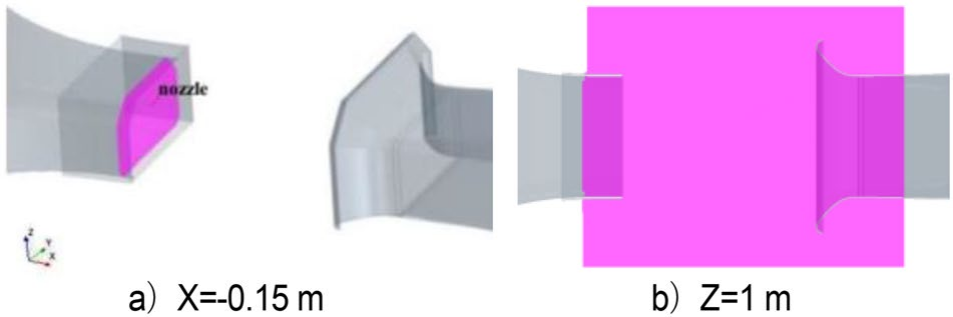
The layout of the sampling plane.

**Figure 5:**
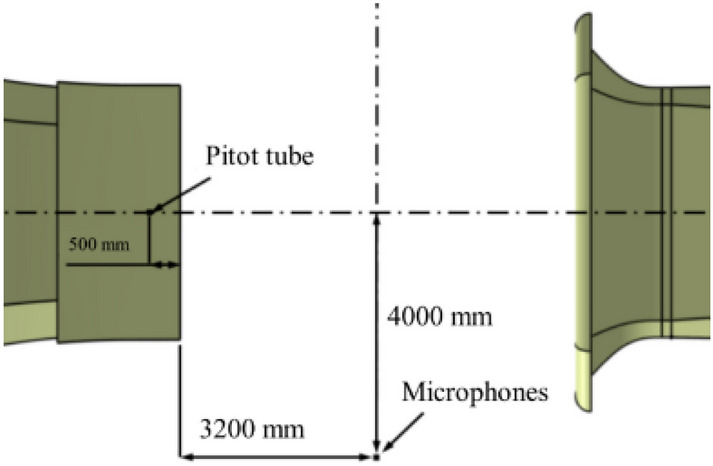
The layout of the sensor.

The center of the nozzle plane is taken as the coordinate origin. Monitoring points are set at the inner of the nozzle at x =  − 0.15 m, and y = z = 0, to simulate the low frequency pressure fluctuation characteristics of the wind tunnel. The XY plane at z = 1 m is taken as the center plane of the flow field, and the flow field characteristic values, such as pressure, velocity, and turbulent kinetic energy, are obtained, as shown in Fig. 4. The acoustic microphones are arranged in the horizontal plane where the central axis of the wind tunnel is located, that is, 1.1 m above the ground of the test section. They are arranged outside the jet range and equipped with a wind shield to prevent the aerodynamic pressure. The transient wind speed is measured by the pitot tube probe installed on the inner of the nozzle, and its vertical distance from the upper wall of the tunnel is 400 mm. The sensor layout is shown in Fig. 5. The sampling frequency of the pitot tube is 10 Hz. Pitot tubes are unreliable for sampling instantaneous wind speeds. However, pitot tube sampling data have a certain tendency to capture the wind speed range where low-frequency pressure fluctuation occurs, so that the more accurate acoustic sampling equipment can collect detailed flow field information when low-frequency pressure fluctuation occurs.

#### Test preparation

Given that no sound absorbent material is found in the wind tunnel test site, the ambient noise level of the wind tunnel must be assessed before the test starts to eliminate the influence of ambient noise on the operating conditions. The test time is in the afternoon, and the weather is clear and rainless. The ambient noise of the wind tunnel is 56 dB (A). The test is conducted in two stages. The test wind speed interval of the wind tunnel is determined. At a 5 m/s interval, the speed fluctuations of each representative wind speed of the wind tunnel are roughly obtained. Combining the test experience and data results, the core wind speed interval is obtained, and the second stage test is performed. In the second stage, fine sampling is conducted at 1 m/s intervals for the core wind speed range to obtain more complete test data. Based on the above methods and test experience, the test wind speed range of this wind tunnel is 0–40 m/s, and the core wind speed range is 15–28 m/s.

### Numerical and computational parameters

**Figure 6:**
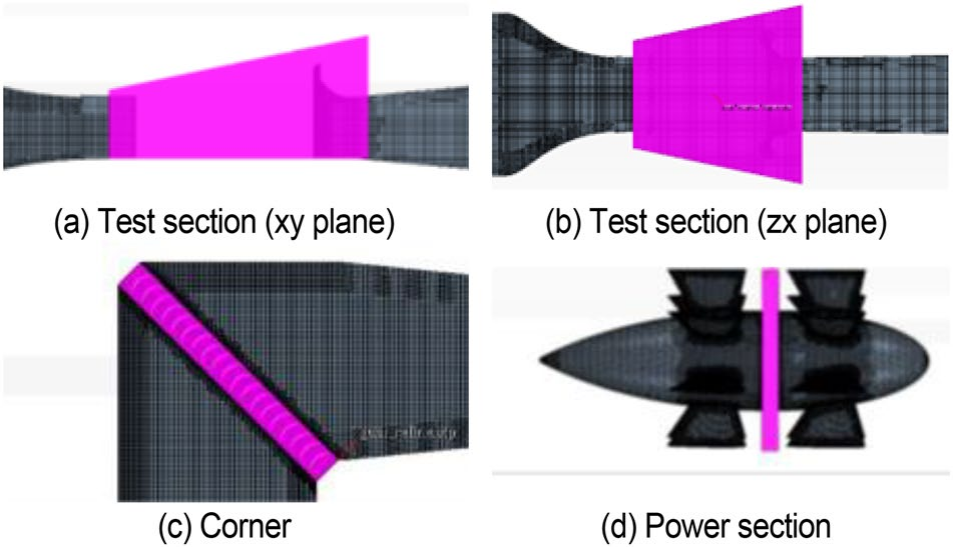
The dense areas of volume mesh.

In this study, a 1: 1 digital model is established in accordance with the wind tunnel of Jilin University. The detailed features are improved to reproduce the low-frequency pressure pulsation phenomenon. The return flow wind tunnel is a closed area due to the plenum chamber structure. The whole wind tunnel is regarded as the whole computational domain, which adopts the trimmer mesh. Five boundary layers are set on the inner surface of the wind tunnel, with a growth rate of 1.2 to accurately simulate the airflow characteristics of the boundary layer in the wind tunnel. The wall boundary function with y +  = 60 is used because the transition of the inner wall of the tunnel is smooth. The boundary layer division of the wall that is not in direct contact with the fluid is forbidden, such as the periphery of the nozzle, the collector, the first diffusion section, and the five walls of the chamber except the floor, to avoid unnecessary waste of computing resources. The dense areas shown in Fig. 6 are established in the test section, corner, and power section, and the 40–80 mm volume grid is adopted to accurately capture the fluid motion state in the test section and simulate the low-frequency pressure pulsation phenomenon.

**Figure 7:**
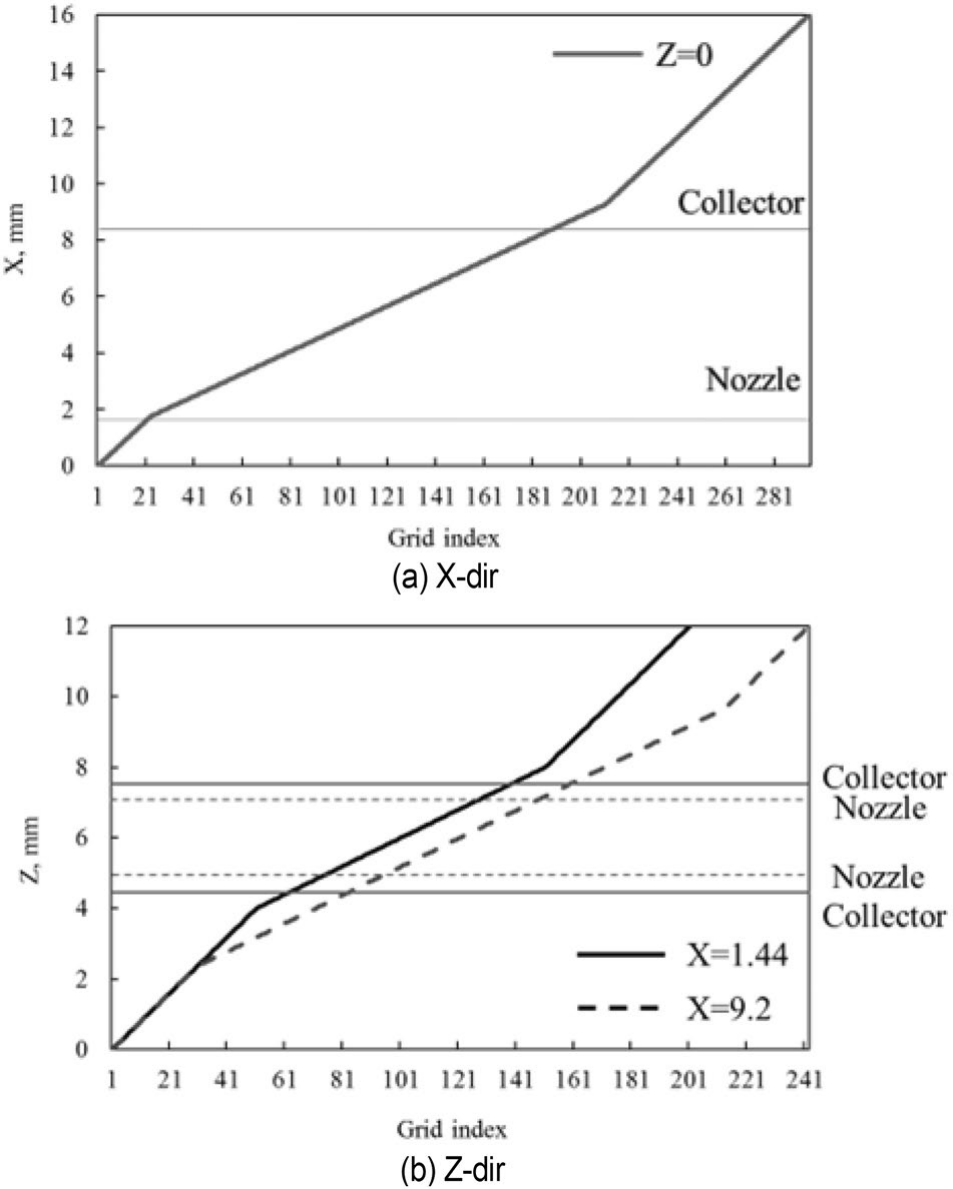
The mesh distribution in the plenum chamber.

Among them, the test section is the area where the mixing layer is easy to occur. Here, the trimmer mesh is also used, but the size is smaller. The mesh distribution in the plenum chamber is shown in Fig. 7.

**Figure 8:**
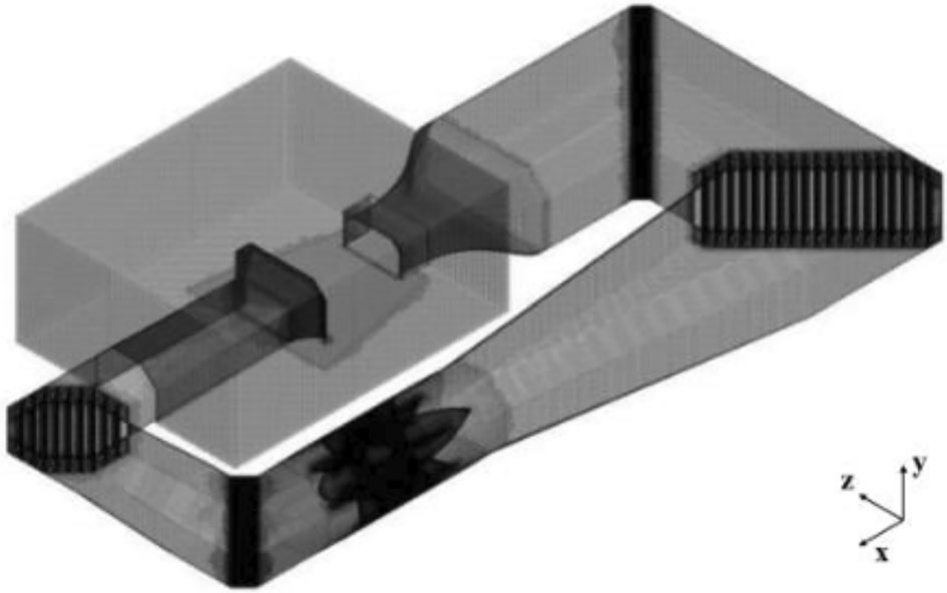
The mesh of the whole wind tunnel model.

The volume grid of the whole wind tunnel model is about 14 million after the verification of grid independence, as shown in Fig. 8.

DES is used as the turbulence model in transient simulation to accurately capture the development and movement of large-scale vortices in the flow field. The unsteady compressible Navier–Stokes equations are solved in rectangular coordinate using SIMPLE algorithm. The perfect gas law closes the system of equations. The equations are solved using the finite-volume method. The convective term adopted the second order upwind. Time-accurate solutions to the governing equations are obtained using the implicit unsteady state scheme. Four time levels are used to perform second-order time discretization. In addition to the above-mentioned spatial discretization and time, the AMG (Algebraic Multi-Grid) method is introduced to pre-process the BiCGStab algorithm to improve the stability and convergence speed of the linear system. Solid and adiabatic wall conditions are imposed to all the wind tunnel. The slip grid method is used to simulate the fan motion. The blades and volume grids of the fan in the rotating domain rotate relative to the calculation domain of the wind tunnel. The rotation speed of the fan is 224 r/min when the velocity at the nozzle exit is 22 m/s. When Initializing flow field, vorticial disturbances non-correlated in the azimuthal direction are added in the boundary layer to create velocity fluctuations at the nozzle exit. Their amplitude reaches the turbulence intensity of 1% at the nozzle exit.

In transient simulation, Courant number is an influence index of simulation analysis, as shown in Eq. (1).

1 $$C=\frac{v\cdot \Delta t}{\Delta x},$$

where Δ*x* represents the minimum size of the divided grid (m). ν represents the convective velocity (m/s). *C* represents the Courant number. The Courant number should be less than 40 for implicit solvers.

The sampling frequency satisfies the Nyquist sampling theorem, as shown in Eq. (2) and Eq. (3).

2 $$\Delta f = f_{\min } = \frac{1}{n \cdot \Delta t}$$

3 $$f_{\max } = \frac{1}{2 \cdot \Delta t}$$

where Δ*f* is the frequency resolution. *fmin* is the minimum frequency. *fmax* is the maximum cut-off frequency. *n* is the sampling length. Δ*t* is the time step.

In this study, the time step Δ*t* = 0.01 s. The total solution time is 40 s, and the data collection is performed in the last 10 s. The Courant number *C* = 5.55, *fmin* = 0.1 Hz, and *fmax* = 50 Hz after considering the above equation, which meets the requirements of simulation conditions.

### Results and discussion

#### Analysis of time-domain and frequency-domain

*Cp* is defined as the pressure fluctuation coefficient, which represents the relative pressure of each point in the flow field. For the flow field with large transient change, *Cp* represents the pressure fluctuation coefficient of the measuring point at a certain instant, as shown in Eq. (4). The dimensionless root mean square value of pressure fluctuation coefficient *Cp*_rms_ is used internationally to characterize the phenomenon of low-frequency pressure fluctuation^29–31^.

4 $$Cp=\frac{\left|P-{P}_{\infty }\right|}{\frac{1}{2}\rho {V}_{\infty }^{2}},$$

where *P* is the static pressure at the measuring point (Pa); *P*_∞_ is the static pressure of far-field incoming flow (Pa); ρ is the density of incoming flow (kg/m^3^); *V*_∞_ is the velocity of far-field incoming flow (m/s).

**Figure 9:**
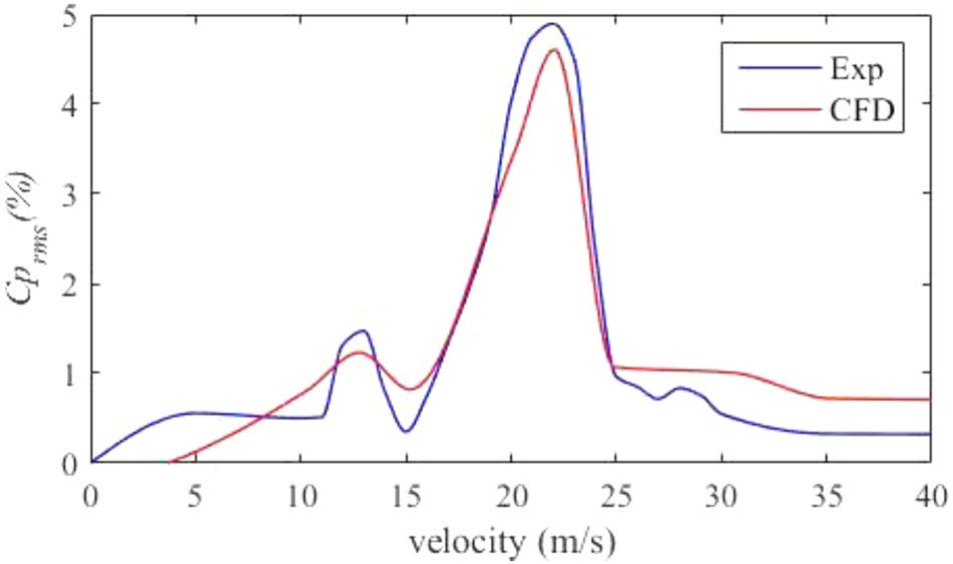
The pressure fluctuation coefficient under each wind speed.

The test wind speed range of the wind tunnel is determined as the common range of 0–40 m/s. The speed fluctuation under each representative wind speed can be roughly obtained at an interval of 5 m/s. In accordance with the test and data results, the core wind speed range is 15–28 m/s. For the core wind speed range, fine sampling is conducted at an interval of 1 m/s to obtain more complete test data. The test data are compared with the simulation results. The obtained pressure fluctuation coefficient under each wind speed is shown in Fig. 9. Uncertainty is an estimate of the magnitude of the error of the physical quantity measured during the test^32^. For indirect measurements q,

5 $$q=\sqrt{\sum_{i=1}^{n}{(\frac{\partial q}{\partial Vi}\delta Vi)}^{2}},$$

where $Vi$ is a direct measurement of physical quantity and *n* is the number of physical quantities. Considering the relative uncertainty of each directly measured physical quantity in the experimental process, the final uncertainty of Cprms is 1.11%.

Figure 9 shows two obvious peaks at 13 and 22 m/s, where the flow field quality of the wind tunnel is poor. Serious low-frequency pressure pulsation occurs at 22 m/s, which has great pulse momentum and widely affects the nearby area, resulting in the bad flow field quality of the wind tunnel in the range of 18–24 m/s. The numerical simulation and test results of wind tunnel pressure pulsation are in good agreement near the dominant frequency. This study only focuses on the low-frequency pressure fluctuation caused by the large peak at 22 m/s.

**Figure 10:**
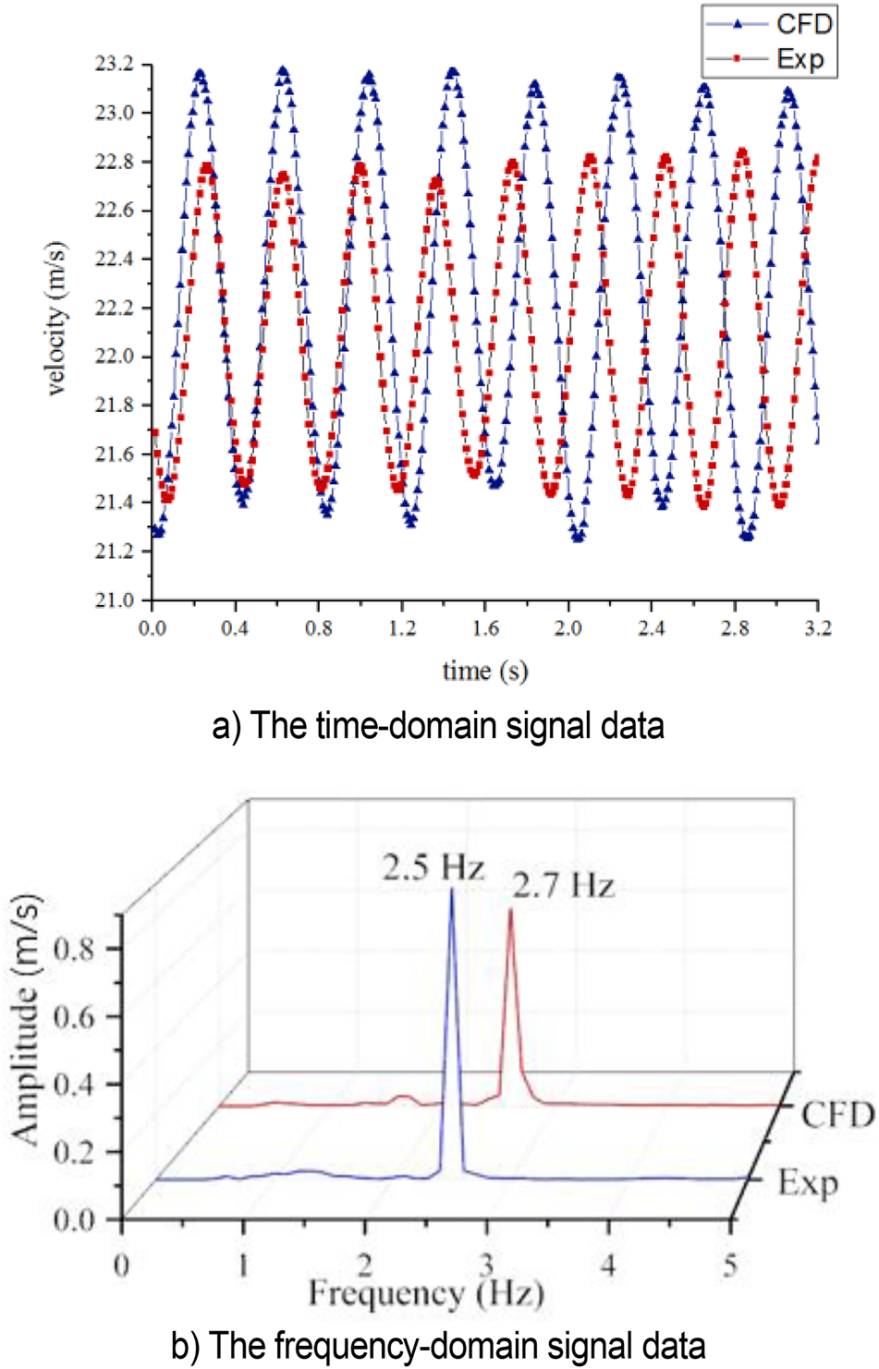
The velocity at the nozzle.

The strong low-frequency pressure pulsation occurs at 22 m/s. The time domain signal simulation under this condition is compared with the test, as shown in Fig. 10. The time domain signal diagram shows that the numerical simulation and test results have a good consistency in describing the pulsation period and amplitude. Under this condition, the velocity has a large fluctuation, with a single peak frequency and a large amplitude of about 1.6 m/s that greatly reduces the flow quality in the wind tunnel. Fast Fourier transform is applied to the time domain signal for obtaining the frequency domain signal graph. The peak frequency of CFD simulation occurs at 2.7 Hz, and the pulsation period is about 0.37 s. The peak frequency of test occurs at 2.5 Hz, and the pulsation period is about 0.4 s. The Strouhal number in the test section is

6 $$Str=\frac{fD}{{U}_{j}}=0.73,$$

where *f* is the peak frequency, *D* is the jet length, *u*_*j*_ is the jet velocity. Audi full-size wind tunnel also reported low-frequency pressure pulsation phenomenon, which occurred at the speed of 27.78 m/s, and the corresponding Str number was 0.78^33,34^. This indicates that the cause of resonance in jilin university wind tunnel is similar to that in audi wind tunnel.

Comparisons of other operating conditions and simulation results in the full wind speed range demonstrate that the transient numerical simulation method can effectively simulate and reproduce the low-frequency pressure fluctuation in open jet and return flow wind tunnel. A small deviation is observed between the simulation and test results. This condition is due to the economy of grids, the omission of components, such as honeycomb and damper net, and the difference between the existing test section and atmospheric connection.

#### Acoustic result analysis

**Figure 11:**
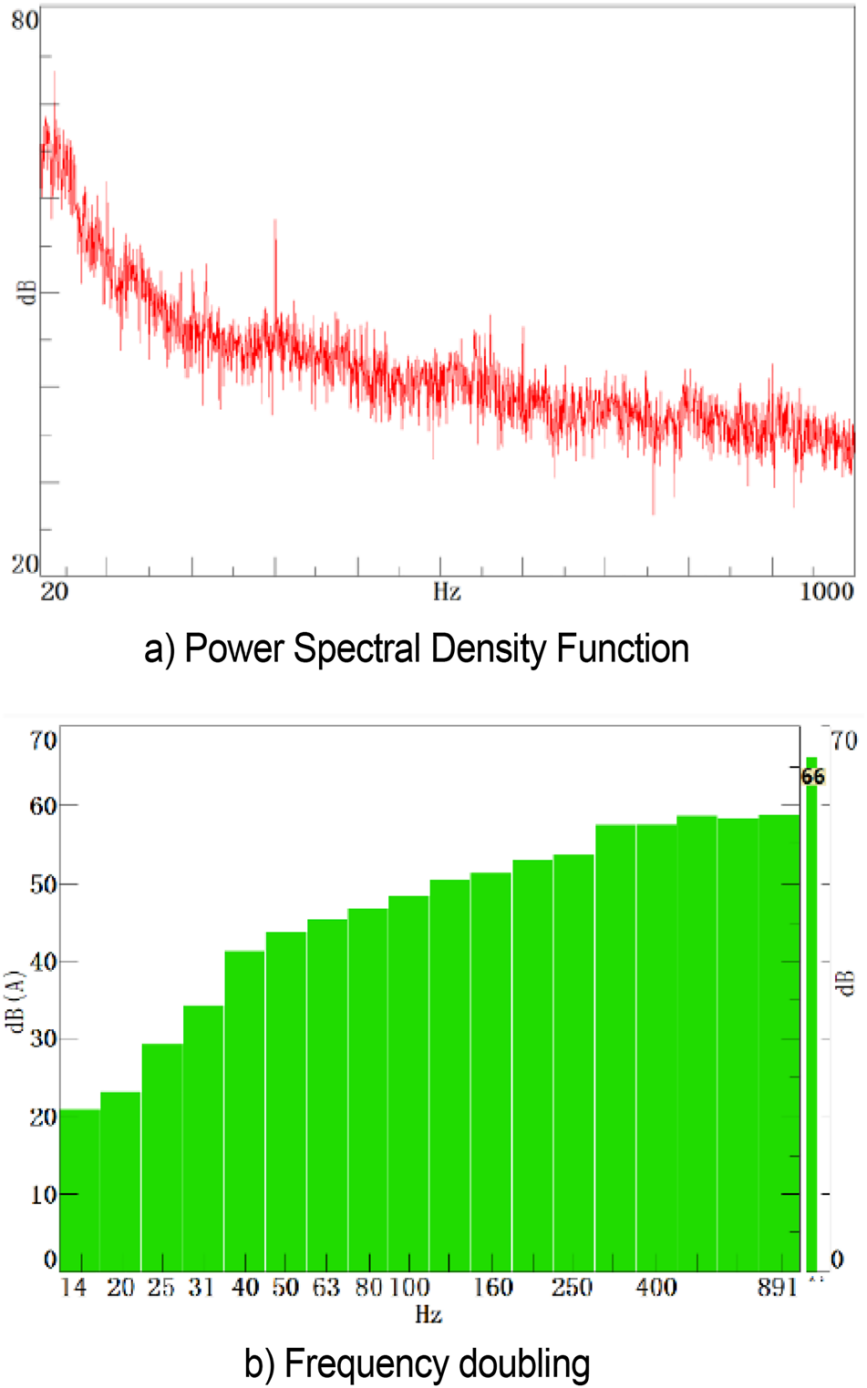
Acoustic measurements at 33 m/s.

**Figure 12:**
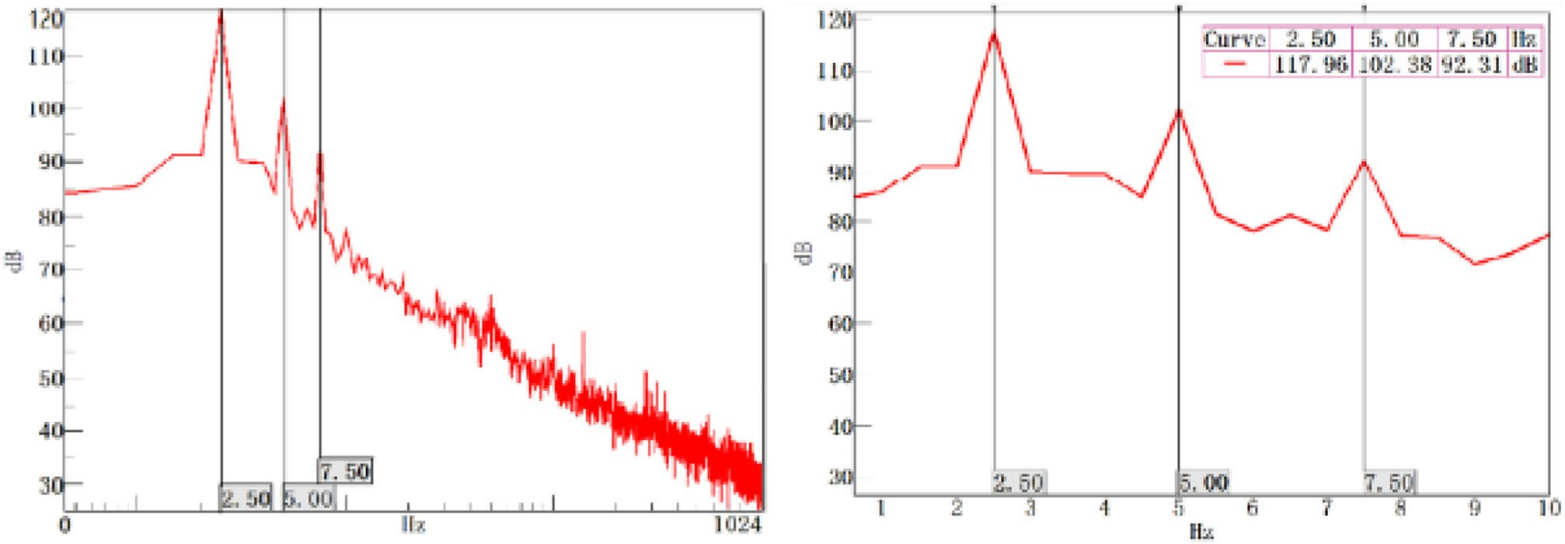
Power Spectral Density Function at 33 m/s.

The time domain signal is processed by digital signal processing. Signal processing parameters include frequency resolution of 0.5 Hz, overlap of 0, Hann window function, power spectral density function, and 50 Hz low-pass filter. Data at 33 m/s operating condition are used to evaluate the acoustic characteristics of the low-frequency area of the wind tunnel, as shown in Fig. 11, in which the power spectrum density is weighted linearly, and the frequency doubling spectrum function is weighted A. From Fig. 11a, the acoustic characteristics of the low-frequency area of the wind tunnel are normal under the normal speed of 120 km/h in vehicle test. With the increase in frequency, the amplitude of the spectrum function decreases evenly without abnormal fluctuation. The wind tunnel of Jilin University is a special wind tunnel for automobile test. From Fig. 11b, the background noise at this wind speed is 66.24 dB (in accordance with the normal vehicle acoustics wind tunnel test standard). The wind tunnel under this wind speed condition has better acoustics characteristics and can be used to perform appropriate vehicle acoustics test. The power spectrum density function at 22 m/s is shown in Fig. 12, which is identical to the flow field fluctuation data.

A large amplitude appears in the extremely low frequency range and corresponds to this pressure pulsation phenomenon. As shown in the right figure, the fundamental frequency of this fluctuation is 2.5 Hz, where the sound pressure level is 117.96 dB. This condition causes multiorder resonance at 5 and 7.5 Hz with the decrease in intensity.

**Figure 13:**
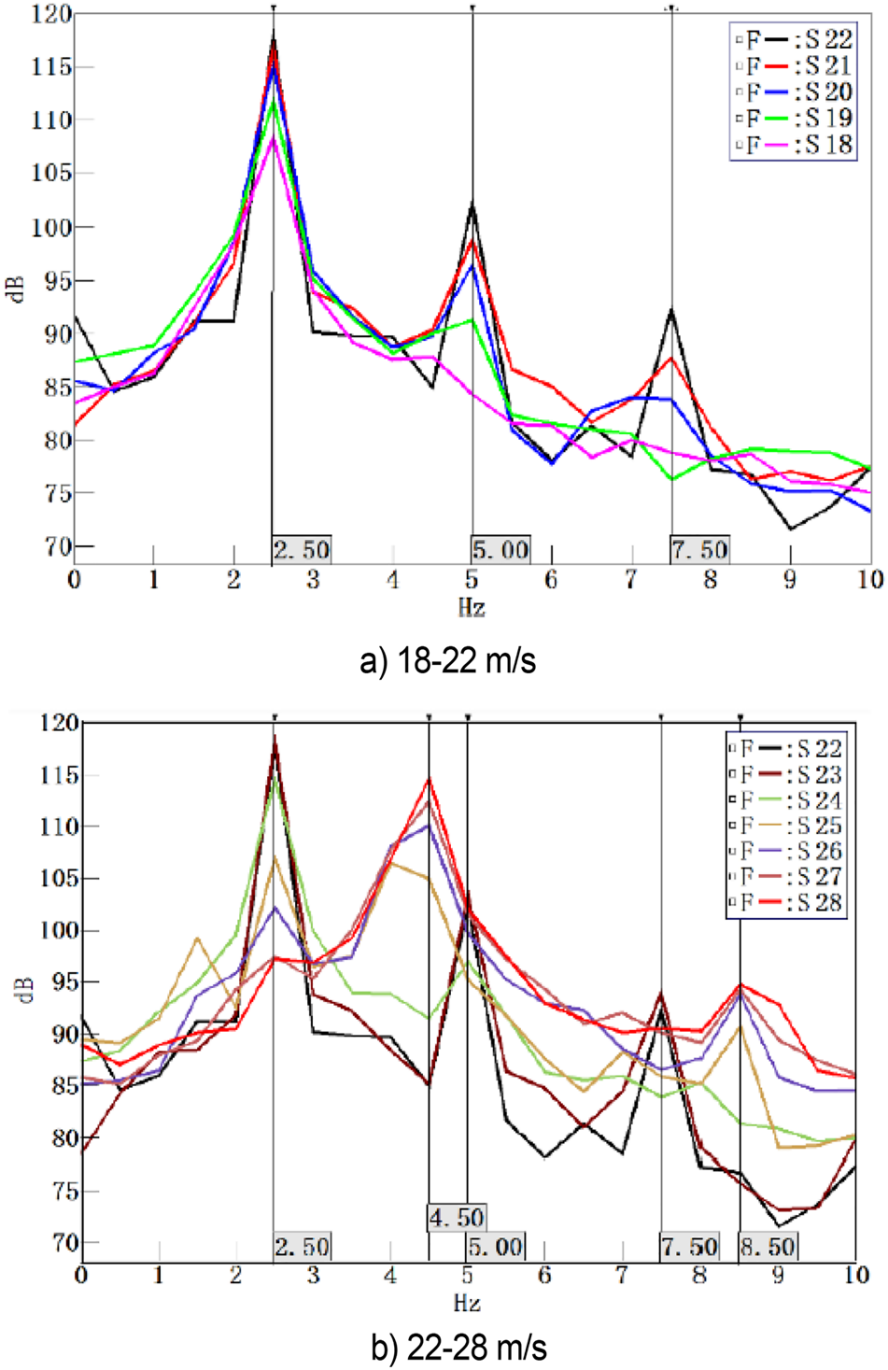
Power Spectrum Density Function at Pulsation Interval Conditions.

The 18–28 m/s range is the interval where the pressure fluctuation is more obvious. As shown in Fig. 13a, the fluctuations in the range of 18–28 m/s have peaks at 2.5 Hz frequency. As the test speed deviates from 22 m/s, the amplitude of the fundamental frequency fluctuations decreases gradually, and the amplitude of the resonance frequency decreases gradually. As shown in Fig. 13b, a peak value at 2.5 Hz is observed for some fluctuations within the range of 22–28 m/s. With the increase in speed, the resonance phenomenon caused by fundamental frequency disappears rapidly. At the same time, a new pulsation frequency of 4.5 Hz is generated at 25 m/s. With the continuous increase in speed, the original 2.5 Hz pulsation frequency is gradually replaced, and the amplitude at 8.5 Hz increases gradually. Based on the analysis of the fluctuations in the 18–22 m/s range, the new ripple frequency may induce new resonance phenomena. The uncertainty of measurement results in this test is 0.66 dB.

#### Formation and evolution of vortex structures

**Figure 14:**
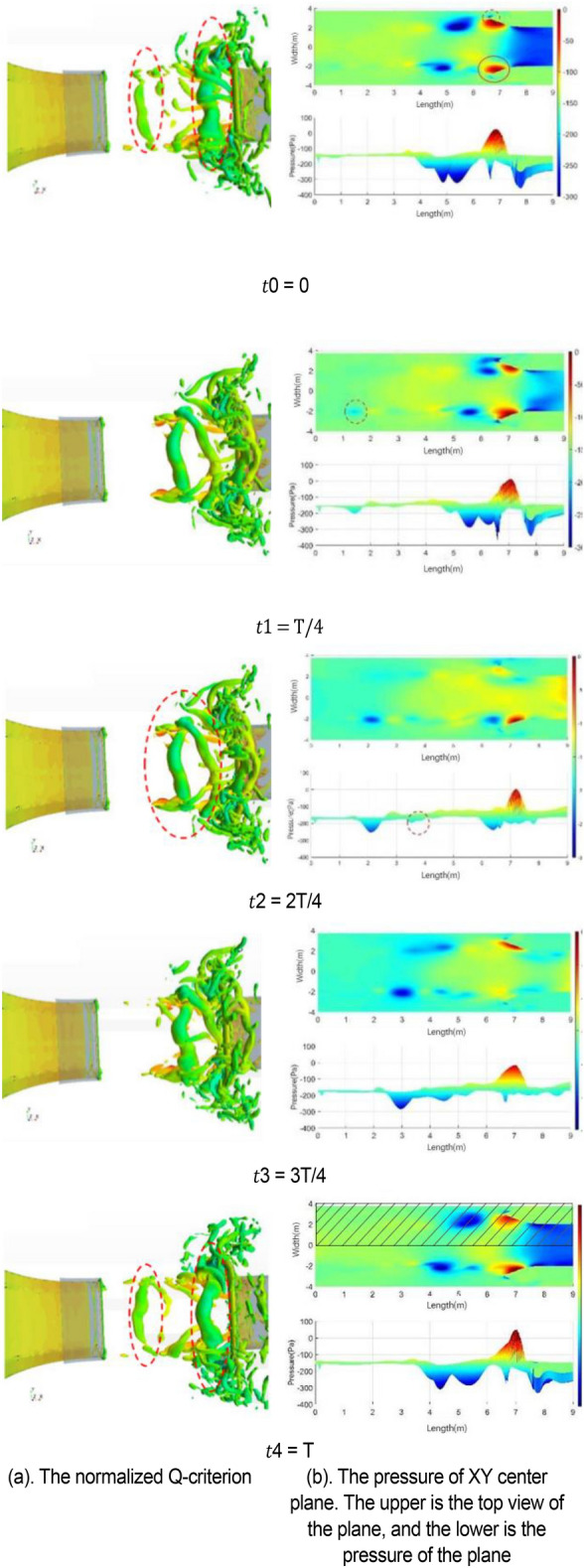
Vortex formation and evolution in the test section.

In the 20–40 s range, the velocity at the nozzle fluctuates obviously around 22.2 m/s. The normalized Q-criterion is taken to show the jet flow field illustrating vortex formation and evolution in the test section in Fig. 14a. The flow field pressure variations are shown in Fig. 14b.

At t0, the nozzle jet layer expands outward in accordance with jet theory. Two vortices are mainly formed. The left-hand vortex has just formed and is only formed by the jet layer at the top of the nozzle. The right-hand swirl has matured. The windward side (solid line area) at the retracted position of the collection port has a large positive pressure due to the close proximity of the vortex structure. Compared with the center of the vortex structure, the differential pressure is close to 350 Pa, and the positive pressure at this point is the maximum during the whole cycle. This positive pressure results in maximum negative pressure at the throat of the collection port, which exceeds the strength of the vortex structure. However, the positive windward side (dashed line area) of the collection port is in a negative pressure state, where the low-pressure vortices are small and strong. From the test section to the diffusion section, the enormous pressure differential changes make the flow field at this time the most blocked state in the cycle. At t1, the vortex at the trailing end of the test section hits the collection port, and the vortex in the middle gradually increases. At this time, the positive pressure area of the collection port is reduced, and the negative pressure in the throat of the collection port is relieved temporarily, but the negative pressure area of the collection port increases because of the impacted of the negative pressure vortex. The flow field in space is extremely turbulent due to the release of the blocked pressure. At t2, the whirlpool hitting the collection port escapes outward and breaks into a small structure. When the pressure at the afterbody is released, the positive and negative pressure zones at the collection port are small, and the flow field is relatively flat. At t3, the new eddy structure matures, and the previously broken eddy structure diffuses further away and becomes more fragmented. At t4, the vortices of the jet layer reach a certain intensity and are screened out to start a new cycle. The intensity of vortices on the two sides reaches a high value, and the regression of flow field is unstable.

From the above analysis, a strong negative pressure area is observed on the windward front of the collection port, which is the part that first contacts the vortices. A strong positive pressure area is found inside the windward side of the collection port. The breaking pressure release of the whirlpool depends on where it hit first. Vortex ring structure may not advance at the same speed. The change in the curvature of the initial vortex structure of the nozzle jet results in uneven self-excitation deformation and 3D structure of the nozzle jet. The eddy structure of the nozzle jet layer first occurs at the top and then at the two sides. The minimum curvature radius of the vortex ring appears on the long axis, and the maximum curvature radius appears on the short axis. Vortex rings with small curvature radius transfer and diffuse downstream faster due to self-induction. Thus, the stub shaft develops at a faster rate, resulting in a “shaft shift” from the outlet to the positioning position. The crushed eddy current still forms a smaller vortex structure in the form of a cyclone, which diffuses outward and absorbs more fluid after the cyclone impinges on the collector.

**Figure 15:**
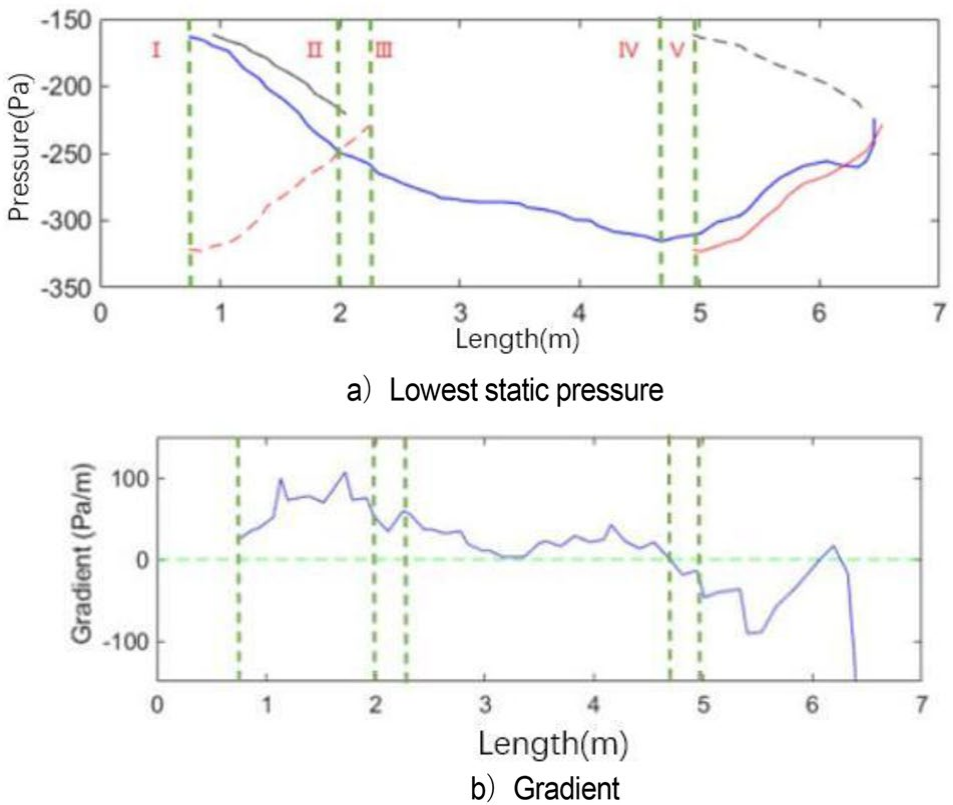
Evolution of vortex (The blue line represents the "current vortex", the red line represents the old vortex, and the black line represents the new vortex. The solid line represents the actual vortex structure in the current period. The red dotted line represents the old vortex which is moved to the timeline of the "current vortex". The same goes for black dotted lines. The green dotted line represents the key time node of vortex evolution in the current cycle).

Figure 15 shows the change in vortex causing pulsation in a complete cycle, including the generation, development, attenuation, and fragmentation of vortex. Along the flow direction, although the left and right sides of the wind tunnel nozzle are geometrically symmetric, the vortex shedding structures at the nozzle exit on both sides of nozzle are incompletely synchronized. However, the change processes of vortices are the same. The vortex data within y = − 4 m and y = 0 m (shadow region in Fig. 14 t4 = T) are used to analyzed in detail. In Fig. 15a, different colored broken lines represent different vortices, and the values on the broken line represent the lowest static pressure of the vortex along the flow direction in the cycle. The green dotted line represents the key time node of vortex evolution in the current cycle.

The vortex represented by the blue broken line is generated on line I (x = 0.74 m). In the area before this position, that is, the left side of line I, the minimum pressure within the jet range of the nozzle is irregular with the axial distance. Only after line I, the absolute value of the minimum pressure within the jet range increases with the increase in jet distance. Therefore, the vortex is generated at this position. The blue line vortex breaks at x = 6.45 m, and this time is recorded as the end point of the cycle.

In the analysis of Fig. 15, the motion cycle of the vortex overlaps. Therefore, the red solid line is the old vortex remaining in the previous cycle when the blue line is used as the basic analysis cycle. The red solid line is translated to the left of line I to obtain the red dotted line due to the same resolution of abscissa. The new vortex of this cycle (blue line vortex) and the old vortex of the previous cycle (red dotted line vortex) have the same starting time at line I. The red line vortex is broken at line III. Similarly, the new vortex (black dotted line) induces at the nozzle in the next cycle starts to generate when the blue line vortex moves to line V (x = 4.94 m). Its position x = 0.93 m can be captured in the flow field. The black dotted line is translated to the left to obtain the black solid line. At the time of blue line vortex breaking, the black line vortex moves to x = 2.05 m.

**Figure 16:**
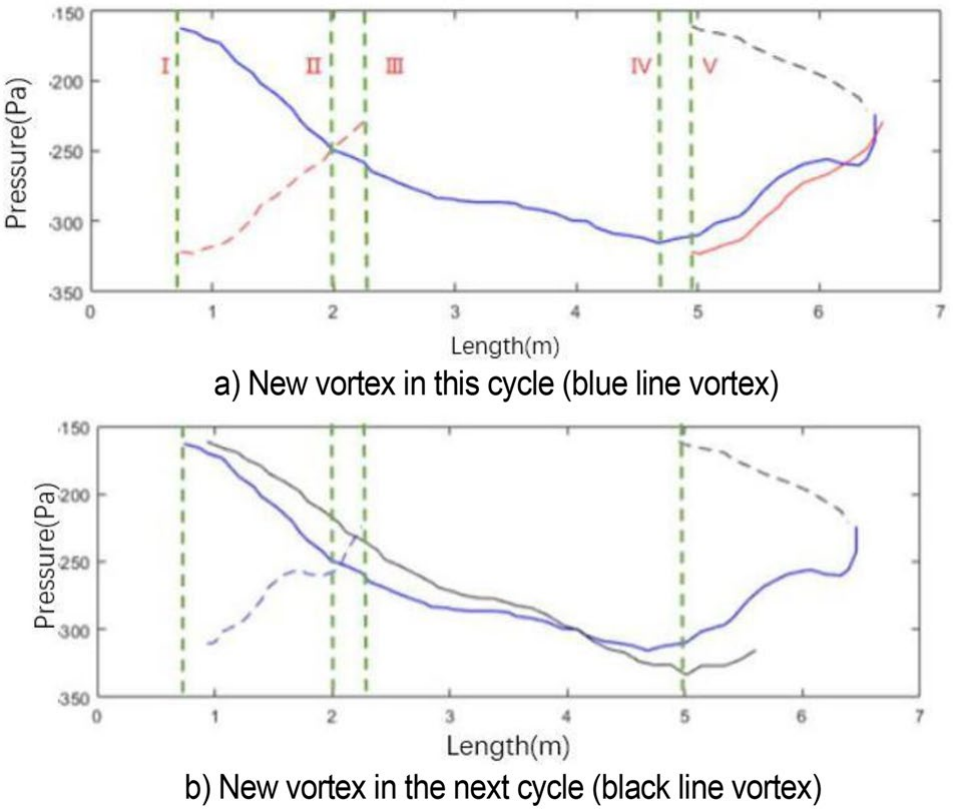
Development process of vortex.

Figure 15b shows the gradient of the lowest pressure of the blue line vortex at each position. The generation, development, attenuation, and fragmentation of blue line vortices are described in detail below. For convenience of expression, “pressure” and “pressure value” mentioned in the following analysis stage refer to the absolute value of the lowest pressure. The generation stage of blue line vortex is shown in Fig. 15. At line I, the pressure value of the red line vortex decreases gradually, and the blue line vortex is generated relatively. The development stage of blue line vortex is shown in Fig. 15 at lines I–IV. The attenuation phase of the blue line vortex is shown in Fig. 15. Under the influence of the windward surface of the collector at x = 6.57 m, the flow field is squeezed. At line IV, the growth rate of the blue line vortex decreases to 0, the pressure reaches the extreme point and begins to decrease, and the black line vortex is induced. As the blue line vortex continues to advance, the negative pressure at the collector begins to ease, and the new vortex (black line vortex) at the nozzle begins to grow. The breaking stage of the blue line vortex is shown in Fig. 15. At x = 6.06 m, the growth rate of the blue line vortex changes from negative to 0, and the pressure reaches a short stable state with a slight rebound. When the gradient of the blue line vortex returns to 0 at x = 6.26 m, the periphery of the vortex is directly squeezed by the collector. Currently, the vortex begins to break. The negative pressure rapidly returns to 0 within 0.02 s, and the gradient value decreases rapidly and reaches the end of the cycle at x = 6.45 m. The gradient value of the vortex is − 485.1 Pa/m (not shown in the figure due to the scale of coordinate axis). A clear understanding of the development process of blue line vortex is obtained through the above analysis. This study mainly explores the generation and fragmentation stages of red line vortex, blue line vortex, and black line vortex. In Fig. 16b, the black solid line is the pressure change in the black line vortex in its own cycle.

The generation position and induction mechanism of vortex can be explained by Fig. 16. The formation position of the blue line vortex and black line vortex at line I is extremely close, and the formation time is the same, which is reciprocal to the dominant frequency of pulsation. Although the vortex structure has been induced at the nozzle, the new vortex structure begins to be cultivated with the attenuation of the old vortex structure at line I. The breaking position and breaking time of vortex can also be explained by Fig. 16. At the end of vortex development, the position of the red dotted line vortex and blue dotted line vortex at line III is the same. This position is caused by the collision of collector, so it is determined by the position of the collector. The vortex breaking time is determined by the resonance frequency affected by jet feedback and pipe modal resonance.

## Control measure

### Simplified model

The low-frequency pressure pulsation is produced by the joint action of jet feedback mechanism and loop resonance mechanism. The former is mainly determined by the jet of the nozzle and the feedback of the collection port, and the latter is mainly determined by the parameters affecting the mode of the pipeline loop. The main body and key components of the wind tunnel are simplified to make it more universal.

**Figure 17:**
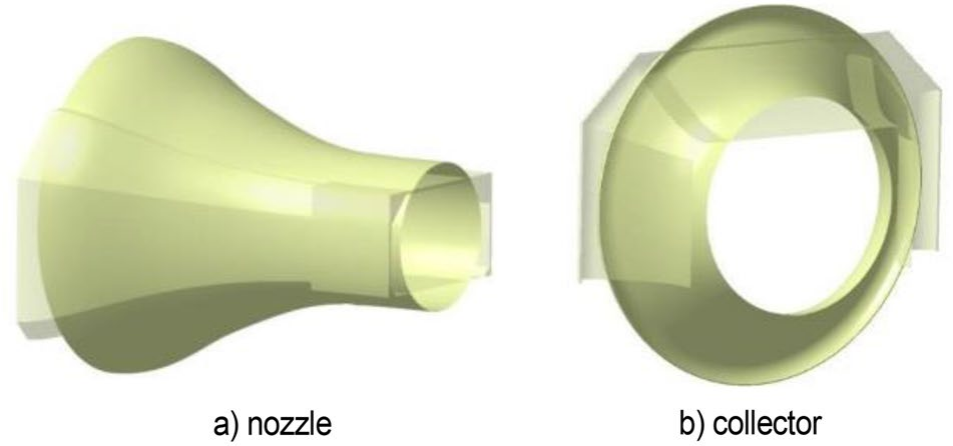
Comparison of some part of the wind tunnel (transparent structure is the original model).

The nozzle and collection port are simplified as Fig. 17. The hydraulic diameter and the length and contraction ratio of the contraction section are kept consistent to obtain a new contraction section and stable section. The remaining main influencing factors are the length and shape curvature of the collection port. The former will affect the length of the test section and the time when the vortex strikes the collection port. The latter will affect the position of the windward side and the contact of the vortex hitting the collection port. The modification of the tunnel body is similar to that of the nozzle. The diffusion ratio and length of each section are unchanged by keeping the hydraulic diameter unchanged, and the simplified model and the original model have flow field similarity by using circular pipes to replace the original polygonal tunnel structure.

**Figure 18:**
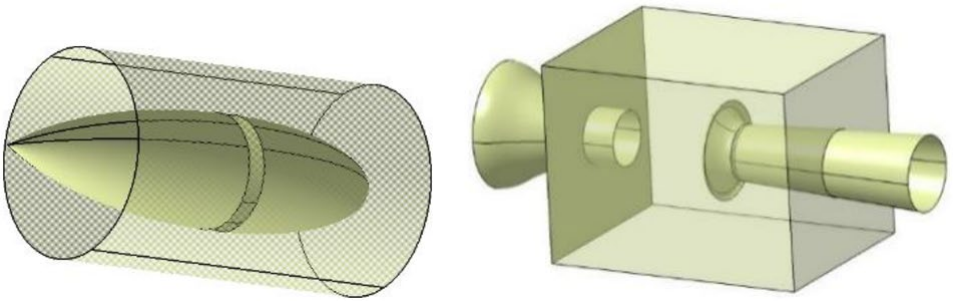
Simplified models of the fan and the chamber.

The relatively complete fan model is used in the original wind tunnel simulation analysis, and only the fairing model is retained in the simplified model, as shown in Fig. 18. Given that the bottom of the original chamber is connected with the nozzle and collection port, a thick boundary layer is formed when the air flow reaches the ground of the test section. In the modification process of the nozzle, it is changed into a circular treatment to further highlight the jet feedback phenomenon and eliminate the influence of the boundary layer effect. For the same reason, the connecting layer between the chamber and the ground is removed. In the case of roughly the same volume of the chamber, the bottom plane of the chamber is moved down to make all the nozzles in an isolated state, so as to ensure the uniqueness of the jet at the nozzle.

**Figure 19:**
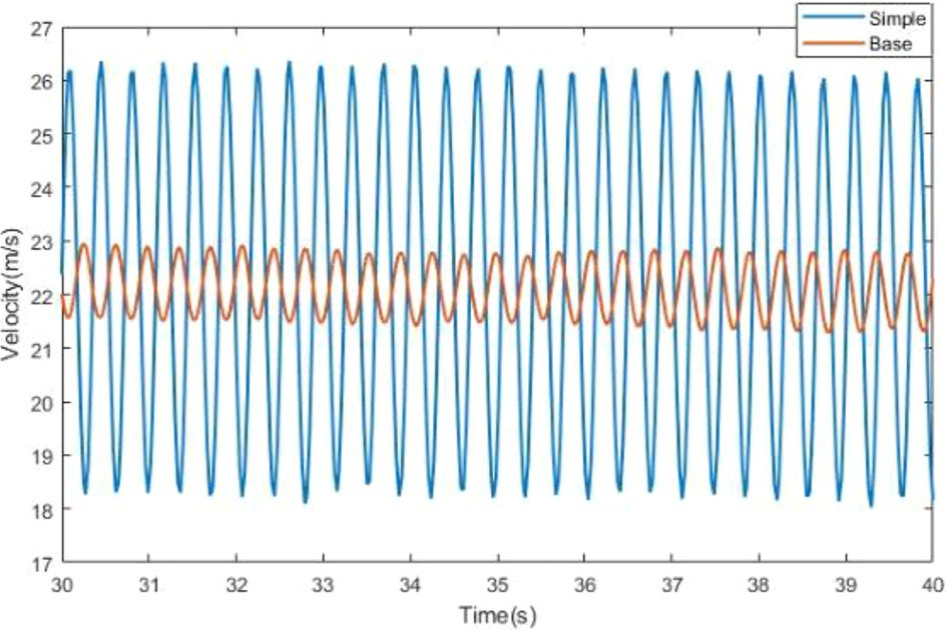
Time domain of simplified model (the blue line is the simplified model and the red line is the original wind tunnel model).

Figure 19 compares the velocity fluctuation in the time domain of the simplified model. Table 1 shows the characteristic parameters of the simplified model and the original model. The simplified model has the phenomenon of low-frequency pressure pulsation, where its amplitude is many times that of the original wind tunnel, and the frequency is highly consistent with the pulsation frequency of the original wind tunnel. This simplified method retains the frequency of low-frequency pulsation in the original wind tunnel and amplifies the phenomenon, which is conducive to the further study.

**Table 1:** The characteristic parameters of power spectral density function.

Model type	Frequency (Hz)	Amplitude (Pa^2^/Hz)
Simplified model	2.75	73.5
Original model	2.76	4.3

**Figure 20:**
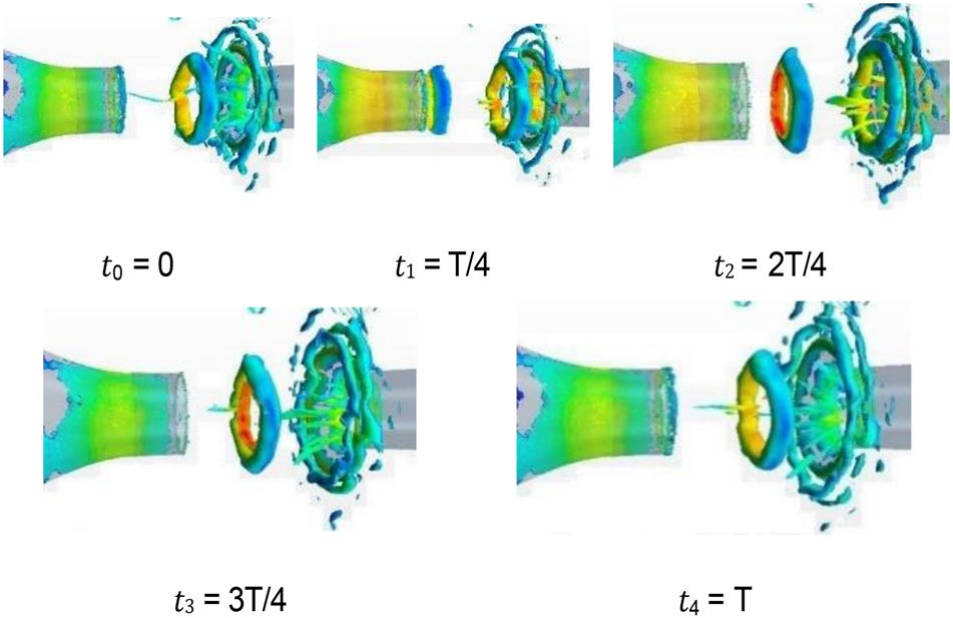
The isosurface cloud diagram of Q criterion of the simplified model (Q = 200 /s^2^).

Figure 20 shows the isosurface cloud diagram of Q criterion in one cycle of the simplified model. The basic characteristics of the flow field of the simplified model are the same as those of the original wind tunnel, but clearer and simpler. The vortex structure of the nozzle jet is complete because the nozzle is circular. The forward speed of different positions of the vortex ring is the same, thereby avoiding the axis transformation of the rectangular jet and facilitating the follow-up study.

**Figure 21:**
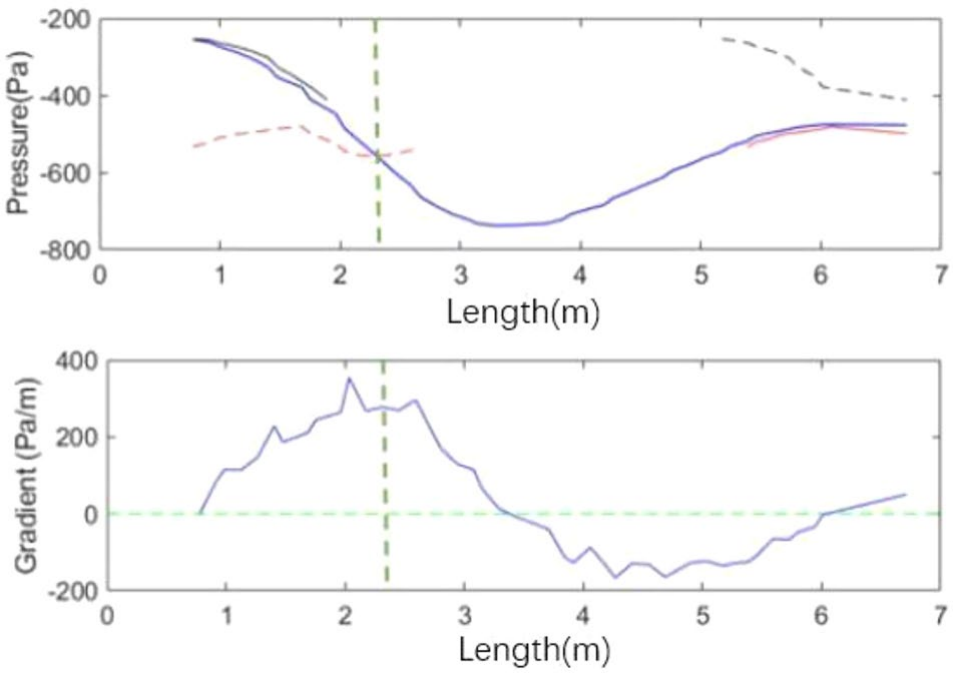
Vortex pressure variation curve (the green vertical line on the right represents the time when the pressure values of red line vortex and blue line vortex are equal).

Figure 21 shows the change process of a single vortex in the simplified model. The pressure change curve of the simplified model illustrates that the overall pressure still decreases first and then increases. The new vortex maintains a rapid growth before the two vortex pressures are equal, and the growth rate of new vortex pressure decreases after the two pressures are equal. The fitting of red line vortex and blue line vortex, red line vortex and black line vortex in the simplified model is better than the original model, showing that this simplified method can ensure higher similarity between cycles.

The difference between the simplified model and the full-scale model is that the growth rate of the simplified model is high, reaching the extreme value of the pressure value in advance and maintaining for a certain time. The simplified model completely shows the three stages of vortex development: induced growth, passage, and survival, attenuation, and fragmentation. The comparison shows that the duration of each stage is affected by many factors and is related to the state of flow field. The induction of new vortex has nothing to do with whether the wake vortex reaches the minimum pressure or whether it decays and is affected by the resonance frequency. However, the growth rate of the new vortex is related to the change in the intensity of the wake vortex. No sudden pressure change is observed in the simplified model.

**Figure 22:**
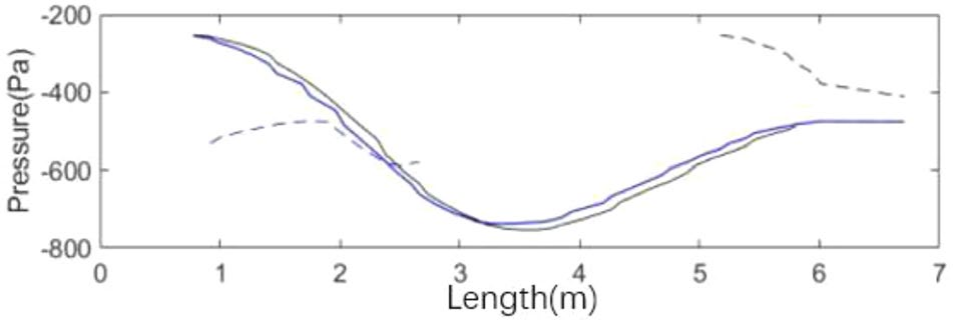
Development of black line vortex.

Figure 22 shows the development of black line vortex. In the simplified model, the development trend of vortices in different periods is highly consistent. The duration of each stage is similar. The induction time and position of black line vortex are consistent with that of blue line vortex, and the development at the end of blue line vortex is the same as that of red line vortex. Compared with the full-scale model, the simulation analysis has stronger periodic consistency, which is convenient for the subsequent modification work.

### Spoilers

#### Digital model

Edge feedback effect is the main excitation to induce pressure pulsation. Nozzle jet phenomenon is a major excitation to induce the edge feedback effect. Therefore, the appropriate intervention of the free jet at the nozzle is conducive to destroy the pulsation law and weaken the pressure pulsation phenomenon. The integrity of the new vortex ring of the nozzle is destroyed, and the pressure pulsation phenomenon is weakened by changing the characteristic shape of the vortex shedding edge of the nozzle when the spoiler is applied to the circumference of the nozzle.

**Figure 23:**
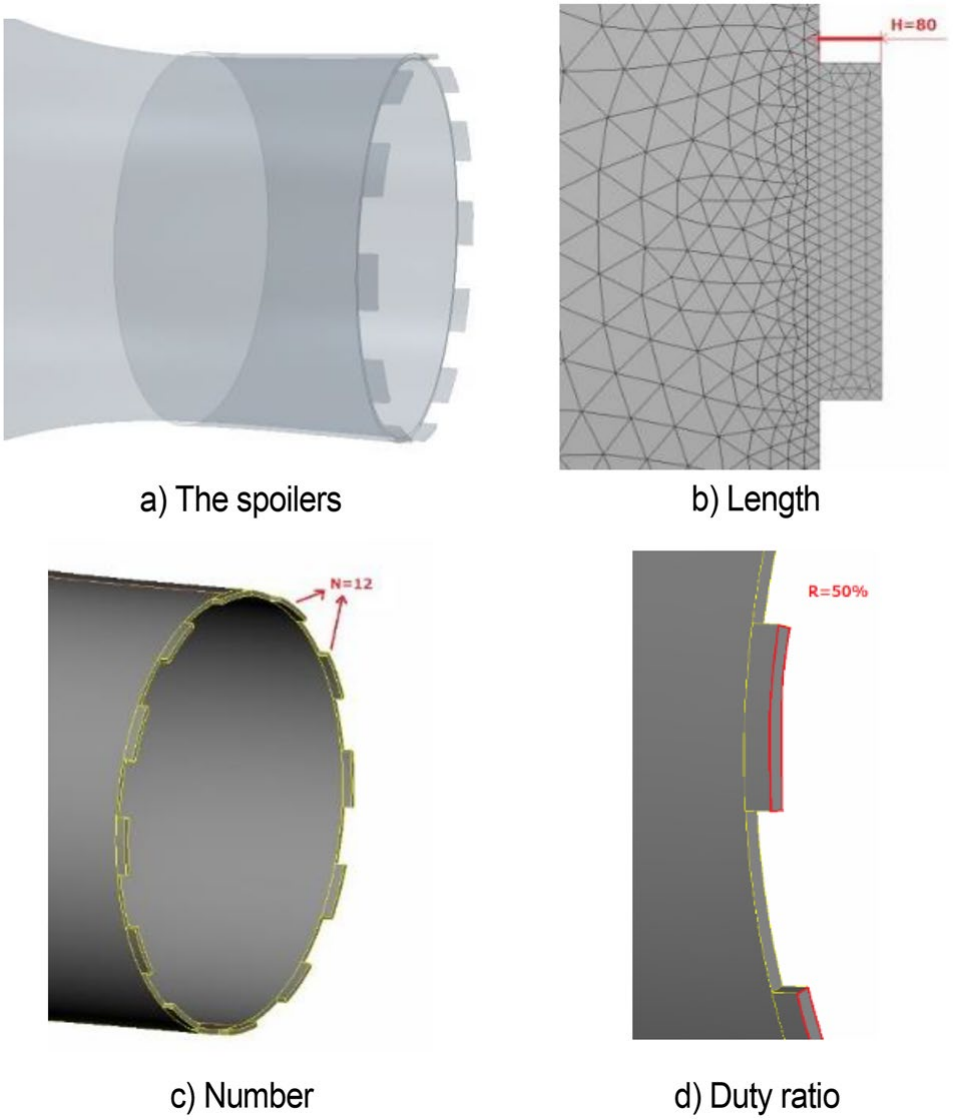
The spoiler model and geometric parameters.

The spoiler model is shown in Fig. 23. Several convex structures are arranged on the nozzle of the simplified model. The geometric parameters of the nozzle spoiler are affected by three factors: (1) the length H (mm) of the spoiler, which represents the length of the spoiler extending out of the nozzle, (2) the number N of the spoiler devices on the circumference, (3) the duty ratio R (%) of the spoiler on the circumference, which is the ratio of the area of the outer surface of the spoiler to the total area of the outer surface of the nozzle. The geometric dimensions of the foundation example are H = 80 mm, n = 12, r = 50%, abbreviated as h80n12r50. To describe the geometry of Spoiler, four layers of meshes are arranged along the flow direction from the nozzle exit to the edge of spoiler. To maintain the aspect ratio of meshes, the meshes along the circumferential direction are the same as those along the flow direction.

#### Results and discussion

**Figure 24:**
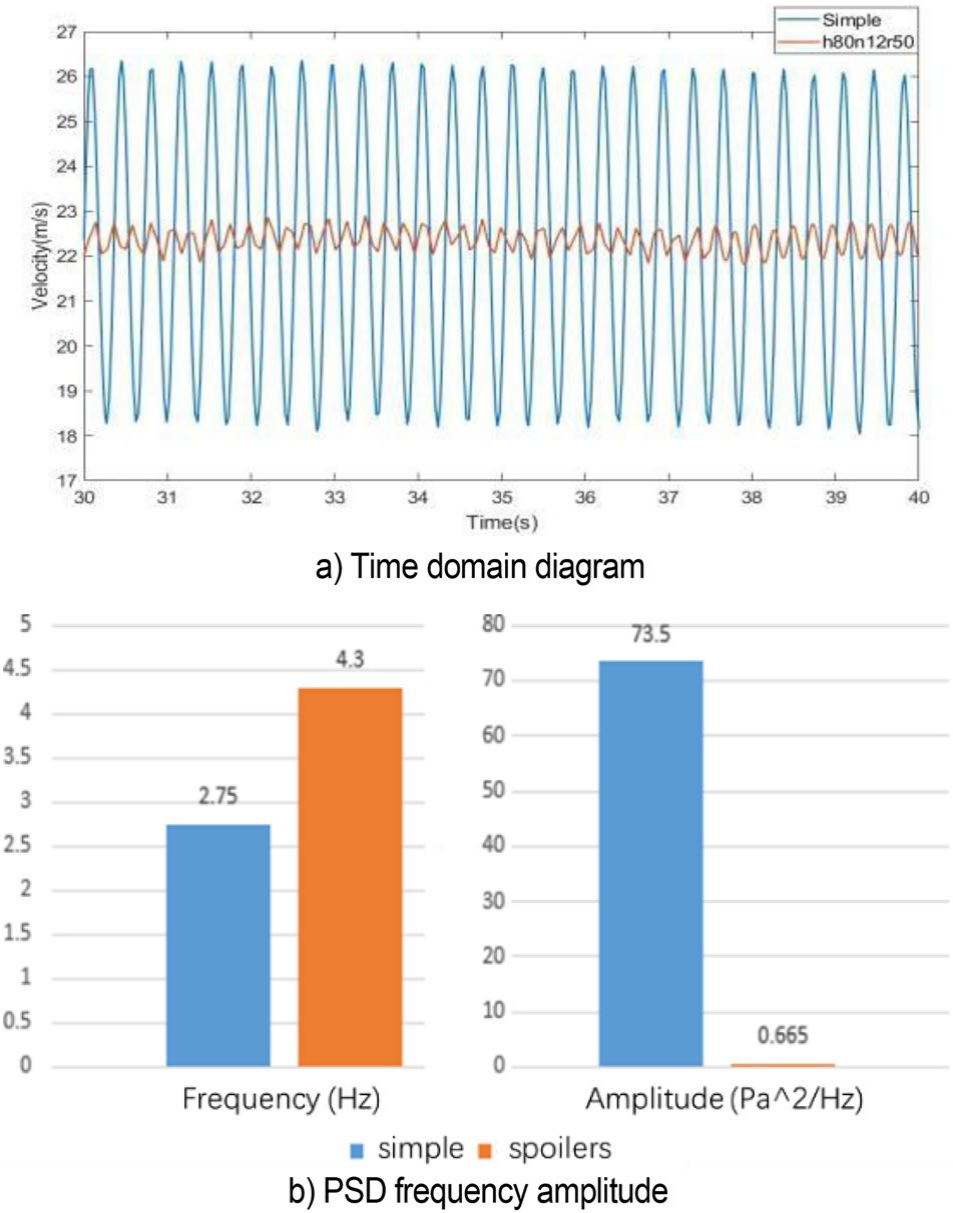
h80n12r50 simulation results.

Figure 24a shows that the spoilers reduce the fluctuation of speed and greatly weakens the low-frequency pressure pulsation in the simplified model. The value of power spectral density function obtained by fast Fourier transform is shown in Fig. 24b. The spoilers increase the main frequency of velocity pulsation to 4.3 Hz, thereby avoiding the resonance interval of the pipeline and do not cause new pulsation phenomenon.

**Figure 25:**
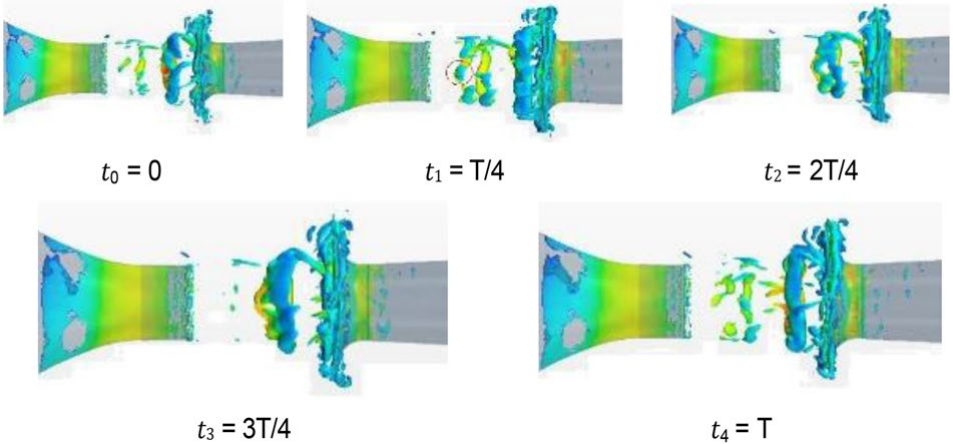
The isosurface cloud diagram of Q criterion with spoiler (Q = 200 /s^2^).

**Figure 26:**
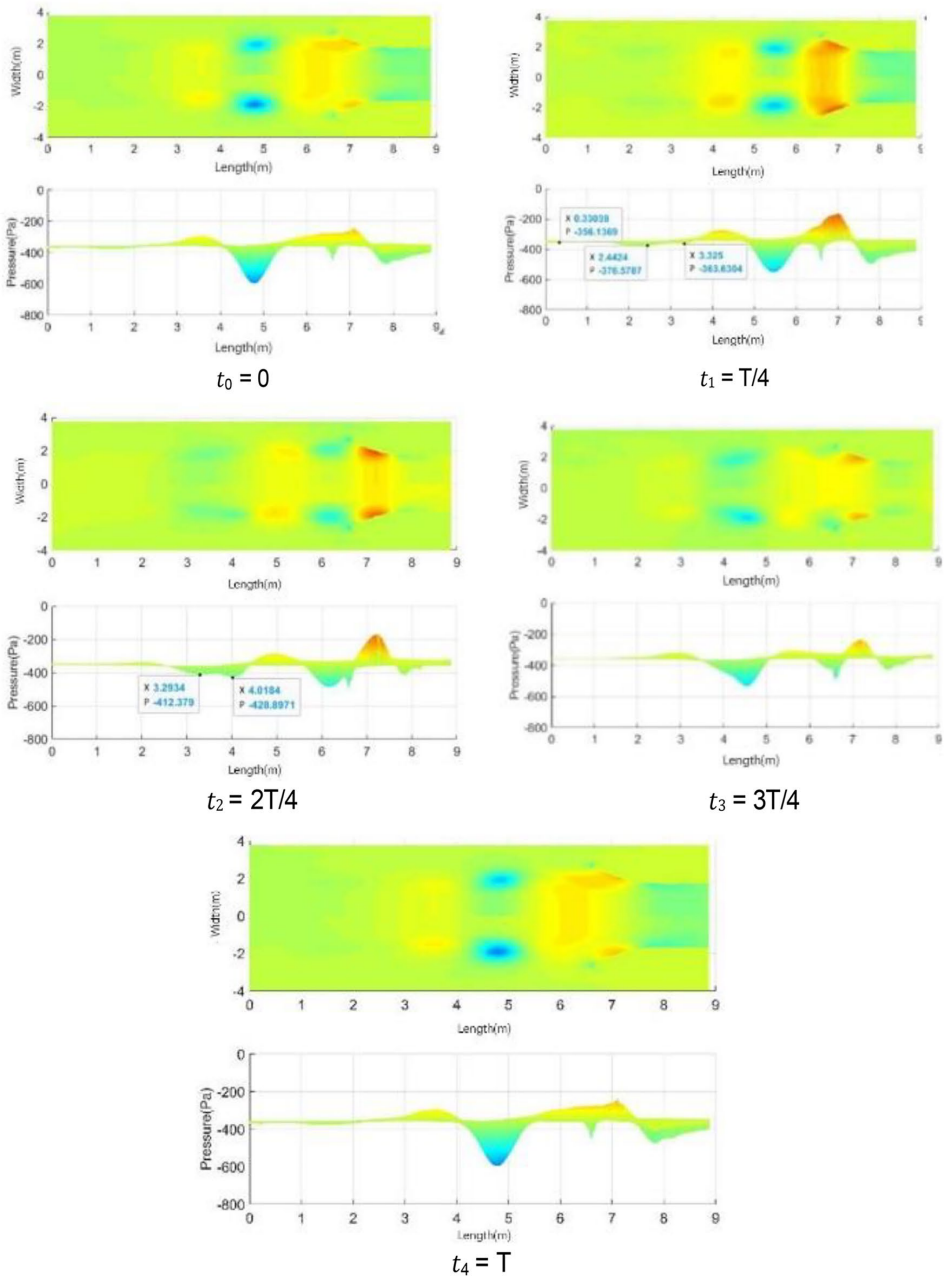
Pressure cloud diagram of center plane with the spoiler.

Figure 25 shows the isosurface cloud diagram of Q-criterion of the model equipped with spoiler in the test section. Figure 26 shows the pressure cloud diagram of the XY plane of the spoiler. The period T is different from that in the simplified model, where T = 1/(4.3 Hz) = 0.23 s. However, T = 1/(2.75 Hz) = 0.36 s for the simplified model. Compared with the basic model, the size of the vortex ring with the spoiler decreases in the whole pulsation cycle. An obvious double ring structure is observed, and the pressure extreme value is lower than that of the original model. At t1, the old vortex ring of the last cycle is about to hit the collection port. The two new rings are incomplete, forming a complementary relationship to a certain extent. At t2 and *t*3, the old ring is broken, the rear ring of the new ring gradually catches up with the front ring, and the latter gradually integrates into the former, forming an inclined ladder pressure distribution. At *t*4, the two rings are completely unified, and the complementary double rings in the next cycle appear. The natural jet phenomenon at the nozzle occurs, and a new vortex structure is produced. Although this phenomenon cannot be eliminated, the frequency of the edge feedback effect can be changed to avoid the modal frequency of the pipeline, which is equivalent to changing the excitation source in the resonance phenomenon, so that the pressure pulsation phenomenon can be well suppressed.

Table 2 lists the pressure values of XZ and XY planes at time *t*1, in which the static pressure of the test section is − 356.14 Pa. For each position with extreme pressure, the pressure values on the two sections are relatively different. At x = 3.3 m, the maximum pressure difference is observed on the XZ section, where a ring chain is found in the front ring. At x = 2.4 m, a ring chain is found in the XY section, which is the position of the rear ring. The extreme pressure value at x = 2 m is the lag position of the rear ring deformation. The scattered vortex ring here is represented by the red area at *t*1 in Fig. 25.

**Table 2:** Pressure values of XZ and XY planes at time *t*1.

Position (m)	Planes	Absolute pressure (Pa)	Differential pressure (Pa)	Weight (%)
3.3	XZ	401.27	45.13	100
	XY	363.63	7.49	16.6
2.4	XZ	356.86	0.72	1.6
	XY	376.58	20.44	45.3
2.0	XZ	385.98	29.84	66.1
	XY	367.32	11.18	24.8

**Figure 27:**
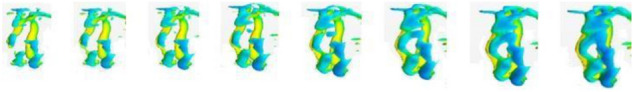
Development of double ring structure from *t*1 to *t*2 ( Δ*t* = 0.01 s).

Figure 27 shows the development of double ring structure from *t*1 to *t*2. With the advancement of the vortex ring, the distance between them continues to shorten. At the same time, each vortex ring slowly fills the gap of its own vortex ring in the YZ plane (the rear ring is more obvious) until the two merge, and all parts of the ring chain are filled.

**Figure 28:**
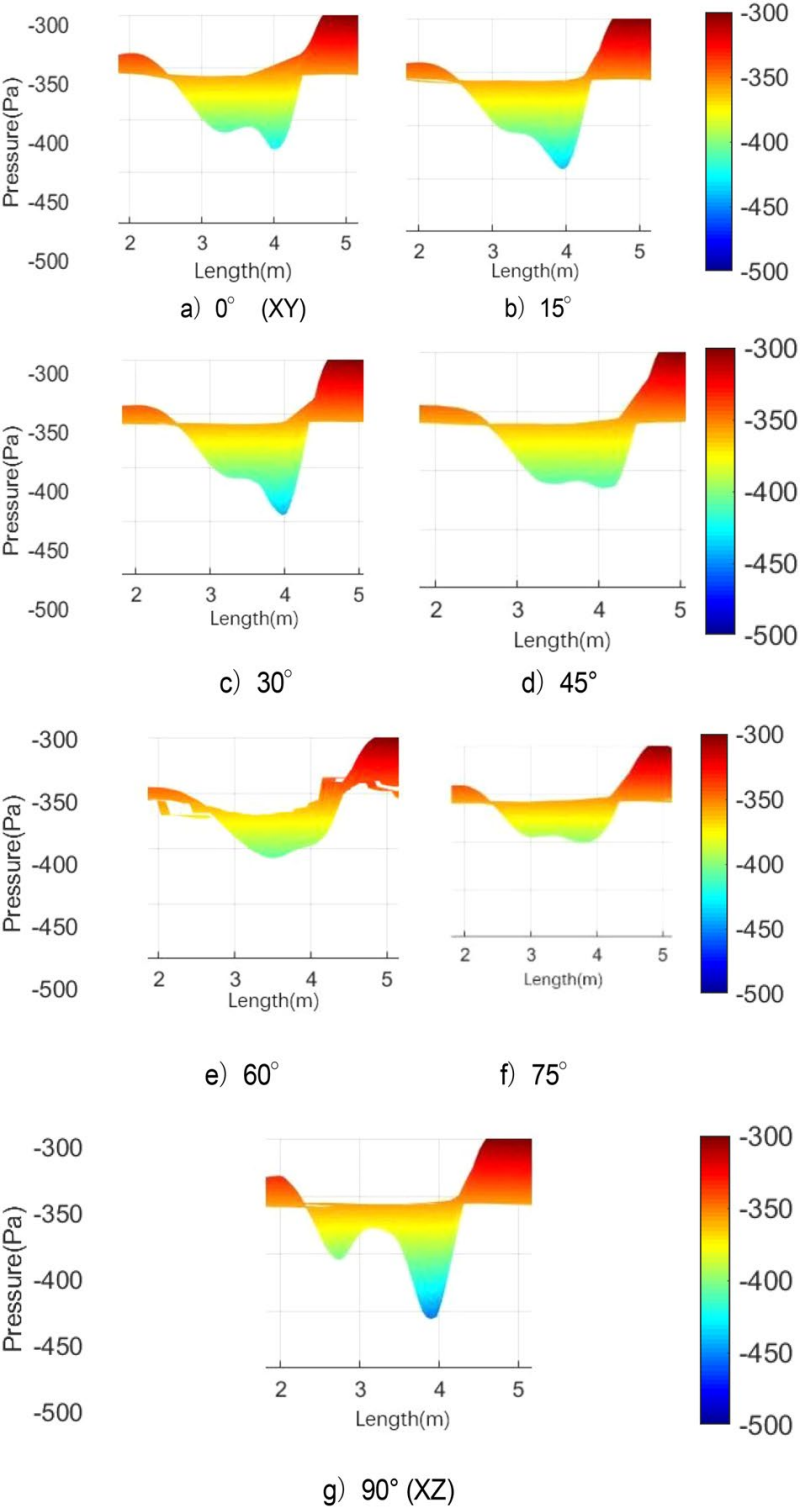
Pressure cloud diagram on different sections at *t*2.

Figure 28 shows the pressure cloud diagram in the interval of x = [2, 5] m on different sections at *t*2. The pressure distribution in this section vary on different sections, and the fusion of each position in the vortex ring is different. Twelve spoilers are found in a circle, that is, each 15° plane is a cross section in the middle of a groove or protrusion. In Fig. 28, planes a), c), e), and g) are the sections where the spoiler is installed, and the rest are the cross sections of the original nozzle. The law of vortex ring fusion is not determined by the existence of spoiler. Although the function of the spoiler is to destroy the original jet structure, the section position without the spoiler may exist in the vortex ring at the front vortex ring. The structural change in flow field is a macro phenomenon, which is caused by the spoiler but not necessarily determined by it. The discontinuity in each vortex ring is related to the geometric size of the nozzle spoiler, but it does not completely correspond.

#### Analysis of influencing factors

The results show that the method of controlling the pressure pulsation by affecting the vortex separation distance of the nozzle has two characteristics. The first characteristic is mutation, that is, this method is sensitive to the change in factors. The effect changes violently, and the regularity is weak. The second characteristic is duality, that is, each modification only corresponds to two situations: effective and ineffective. A situation in which some factors partially alleviate the pulsation phenomenon rarely occurs. The influence of key geometric parameters on pressure pulsation is discussed below.

**Figure 29:**
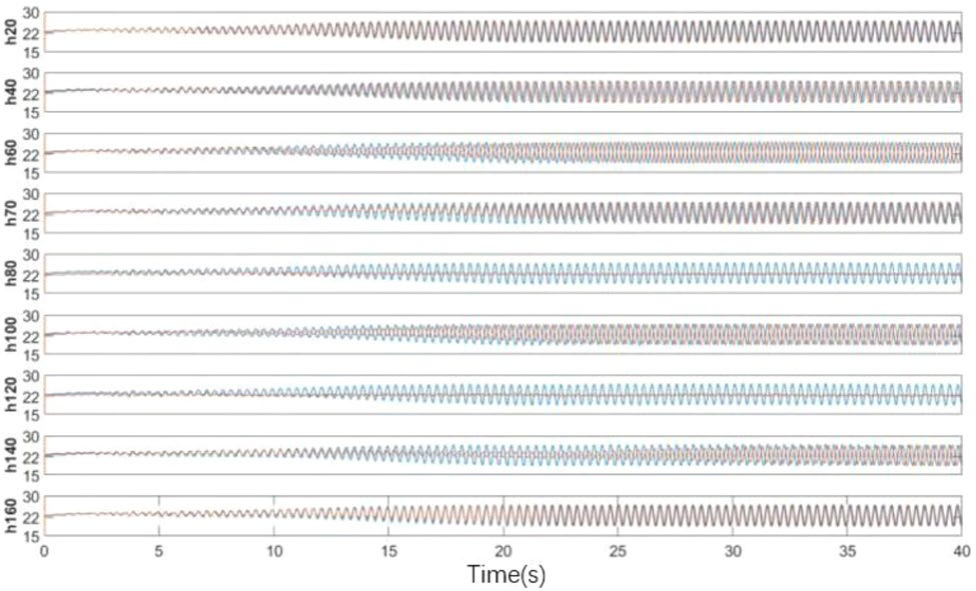
Time domain diagram of length of spoiler (The blue data is the speed of the original simplified model, and the red data is the modified speed curve).

**Figure 30:**
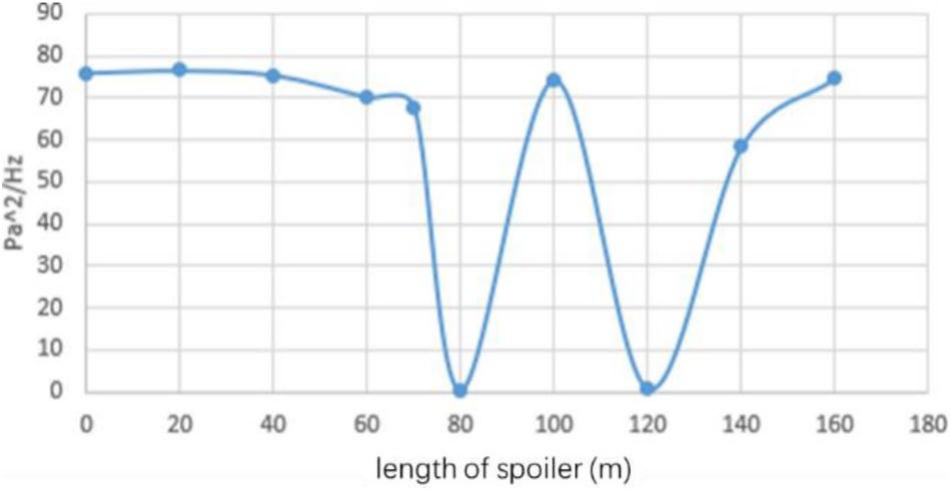
Power spectral density function amplitude.

The length of the spoiler is from 20 to 160 mm, and nine working conditions are set evenly. As shown in Fig. 29, the time domain diagram of speed under various working conditions shows that each condition reaches a stable state in the later stage, and the results of h80 and h140 are considerable. The stable data are taken to obtain the power spectral density function value, as shown in Fig. 30. In h80 and h120, the pulsation phenomenon is greatly alleviated, the effect is obvious, and the effect of other working conditions is poor.

**Figure 31:**
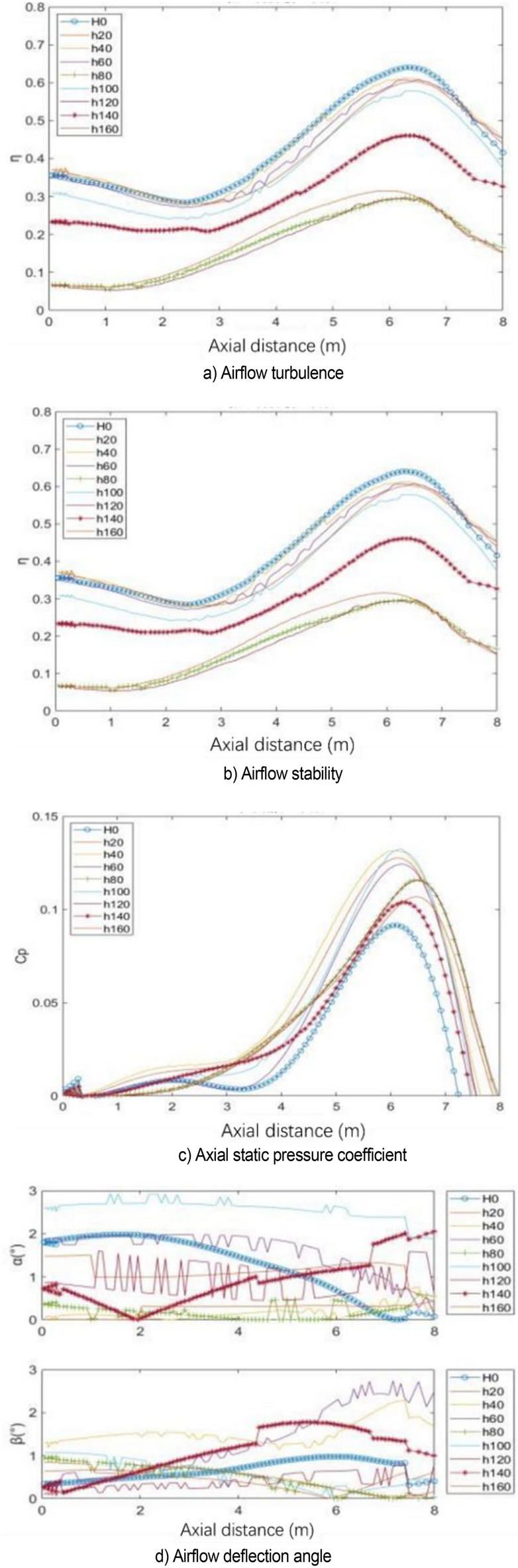
Flow field evaluation index.

The center line of the wind tunnel test section is taken as the detection line, and the flow field evaluation index curve under various working conditions is shown in Fig. 31. The specific value of flow field quality is meaningless because the simplified model is used for simulation. Here, only the relative value is used to measure the modification effect of each modification scheme. The original scheme H0 performs poorly in all evaluation indexes. In the modification scheme of H80, the turbulence degree and stability index of air flow are improved because the pressure pulsation is greatly weakened. Although the axial static pressure coefficient is slightly improved, in the main test section (0–4 m), the static pressure fluctuation of the original model is eliminated under H80 working condition, and the axial static pressure curve is relatively smooth. In the evaluation index of air flow deflection angle, this working condition is well controlled in two directions. The stability of H80 is lower than that of the expected turbulence angle, but it can be improved in the evaluation of h140 turbulence angle.

**Figure 32:**
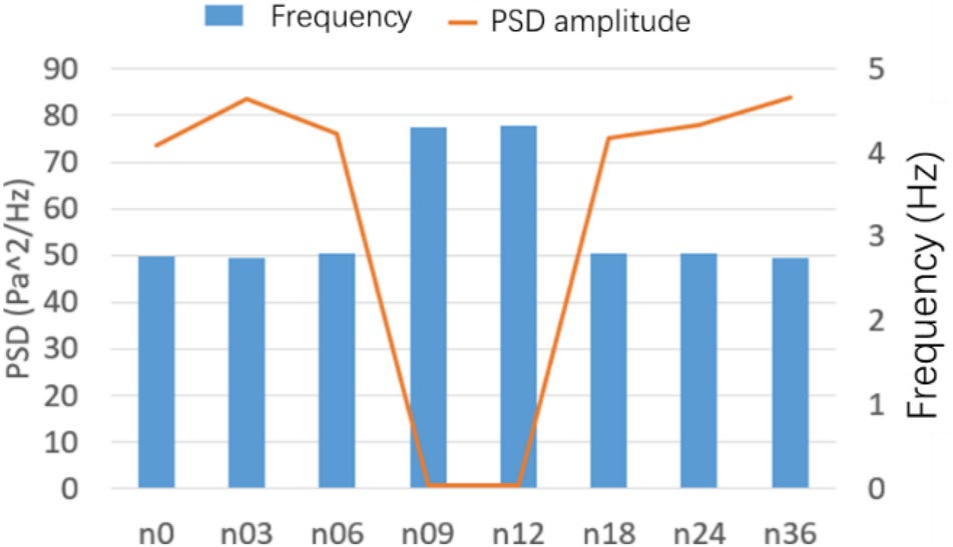
PSD coefficient of number of spoiler.

The number of spoilers on the nozzle circumference is taken as the object, which is transformed into the included angle of adjacent spoiler on the circumference. Figure 32 shows the power spectral density function coefficient of each working condition. The pressure pulsation phenomenon is greatly alleviated under the two working conditions of n09 and n12. The main pulsation frequency of the two conditions is raised to about 4.4 Hz, and the jet frequency of other working conditions is still around 2.5 Hz, that is, the remaining modification scheme fails to disturb the original jet state at the nozzle.

**Figure 33:**
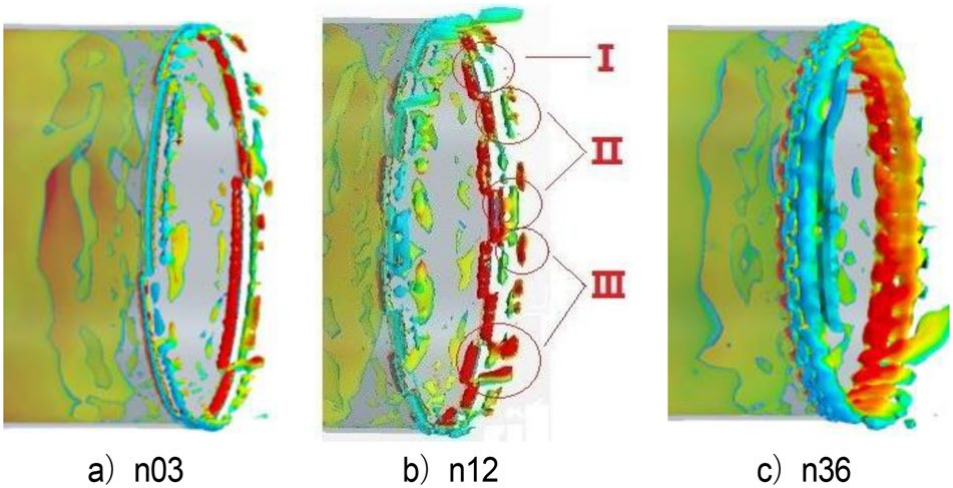
Nozzle vortex structure under typical working conditions (Q = 300 /s^2^).

Figure 33 shows the contour map of nozzle Q criterion under three typical working conditions: n03, n12, and n36. n12 is an effective modification scheme. Different vortex chain structures are formed near the nozzle due to the existence of the spoiler, and they are marked with circles. The vortex chain at I is generated at the nozzle of the original simplified model. The vortex chain at position II is generated by the spoiler, in which the vortex chain above is completely separated from the spoiler, and the vortex chain below is still adhered to the spoiler. The vortex chain at position III is generated at the edge of the spoiler. The difference between it and vortex chains I and II is that it has high speed and is far from the nozzle. The shape above is completely separated from the spoiler, and the vortex chain below is still connected to the spoiler. The vortex structure here is caused by the influence of I and II vortex chains on the air flow outside the nozzle boundary layer. In n36, the type I vortex chain only exists for a moment and directly integrates with the type II vortex chain because the turbulence devices are extremely dense, forming a complete vortex ring structure directly at the nozzle, which does not successfully destroy the jet structure of the original simplified model. n03 working condition is opposite to n36 working condition. Although the three forms of vortex chains exist in a similar manner, the form of each vortex chain is incomplete and the energy is weak. The edges of vortex chains I and II are blurred due to the sparse spoiler and do not cause great damage to the vortex ring structure of the original simplified model.

**Figure 34:**
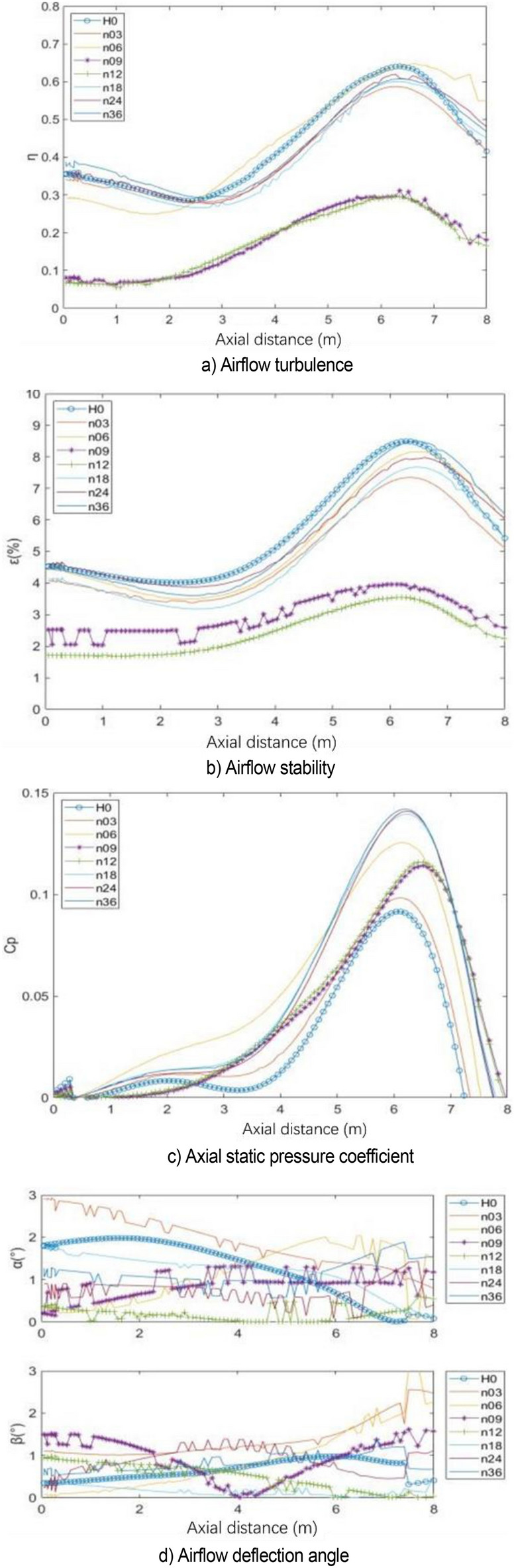
Flow field evaluation index.

Figure 34 shows the flow field evaluation index curve under various working conditions. The effects of n09 and n12 are similar. Each flow field index is improved to a certain extent after greatly weakening the pressure pulsation. Similar to the length parameter, the axial static pressure coefficient increases uniformly after eliminating the fluctuation of the original central section. In the air flow deflection angle index, n09 has some step phenomenon.

**Figure 35:**
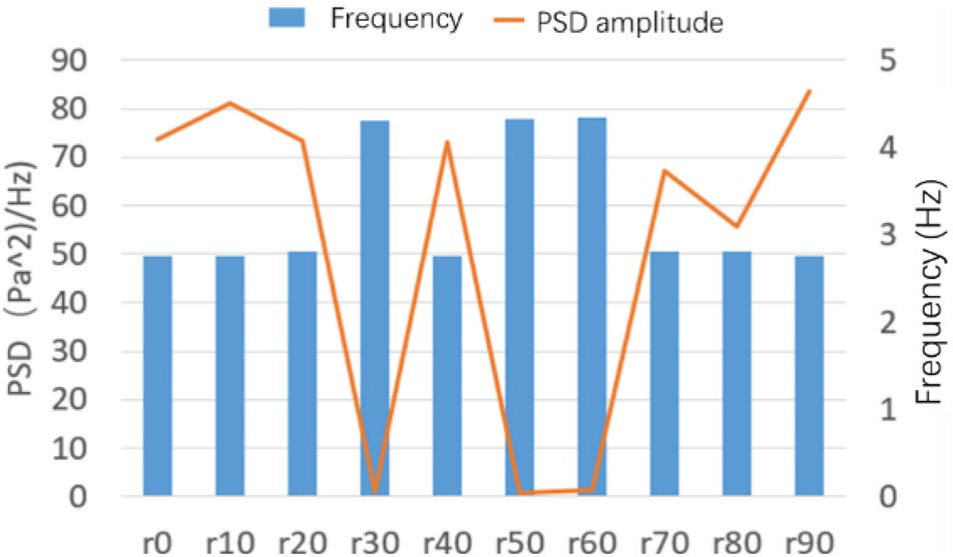
PSD coefficient of duty radio of spoiler.

The duty radio of the spoiler on the nozzle circumference is taken as the object, from 10 to 90%, and a nine groups of working conditions are designed. Figure 35 shows the power spectral density function coefficient of each working condition. Under r30, r50, and r60, the pressure pulsation phenomenon is greatly alleviated. The failure causes of other working conditions are similar to those described above.

**Figure 36:**
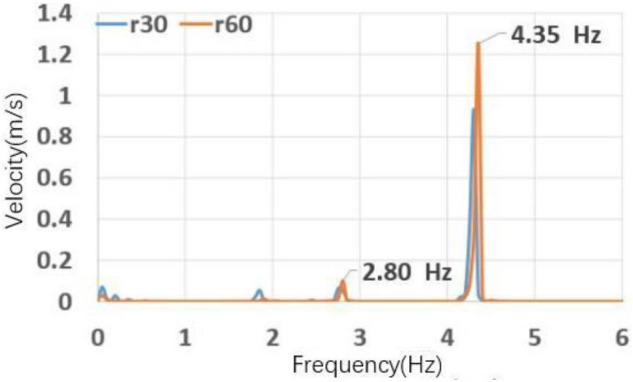
r30 and r60 power spectral density function.

Figure 36 shows the power spectral density function diagram of r30 and r60. The main frequency of the pulsation is 4.35 Hz, and the second main frequency is 2.8 Hz, which is near the original pulsation frequency and contains a certain original pulsation component.

The following suggestions through the further analysis of the influencing factors are provided for the geometric size setting of the spoiler. The spacing of the circular nozzle spoiler is 30°–45°, and the duty cycle is 40%–60%. Extremely large or extremely small of these influencing factors will make the spoiler characteristics invalid. The length of the spoiler needs to be comprehensively judged in accordance with the hydraulic radius of the nozzle and the pulsation induced velocity, which has a certain mutation.

### Flow-follow device

#### Digital model

**Figure 37:**
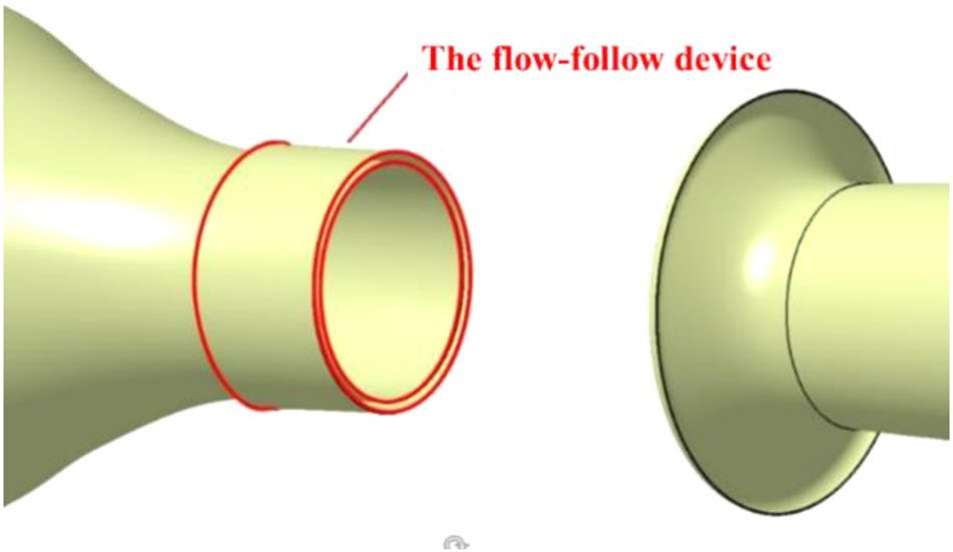
The flow-follow device model.

The air flow begins to separate at the nozzle and tends to diverge outward because the flow field velocity outside the nozzle is lower than that of the inside. The velocity of the outer decreases, thereby providing convenient conditions for the formation of vortex structure. The flow-follow device is set outside the nozzle to mobilize the air flow in the flow field, so that the straight out air flow inside the nozzle has a smaller velocity difference from the outside at the separation point of the nozzle. The separation position of the air flow is transferred outward to the outside of the flow-follow device. The separation characteristics of the shear layer of the jet are changed by the flow-follow velocity. The flow-follow device model is shown in Fig. 37. A new channel is established around the nozzle of the simplified model of the wind tunnel. The new make-up air flow enters the flow field through this channel, damaging the original jet structure.

**Figure 38:**
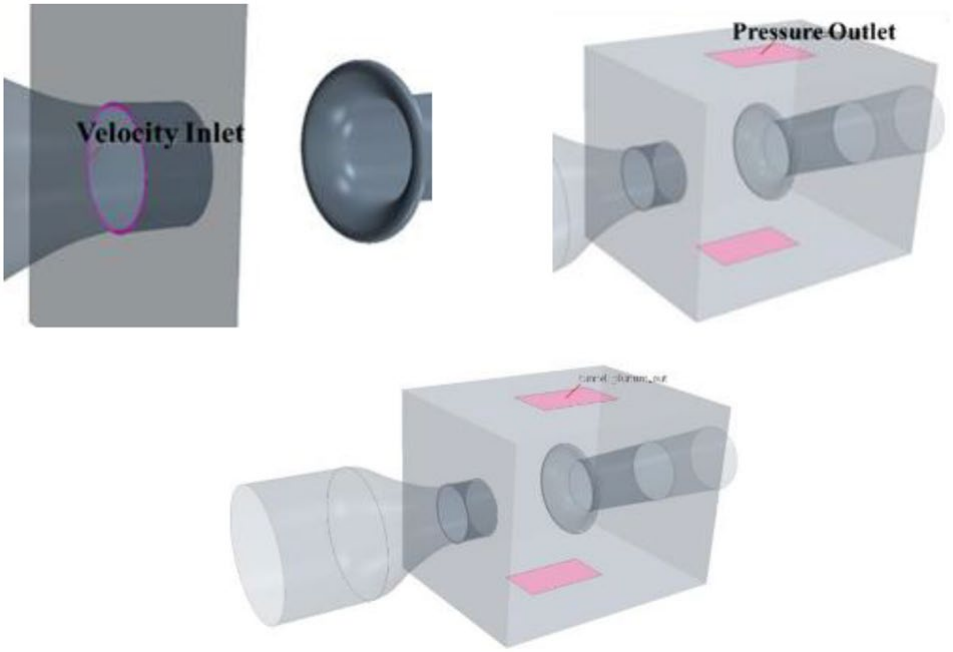
Velocity inlet and Pressure outlet of the flow-follow device.

The solver setting and most boundary conditions of flow-follow device are the same as the former. However, some boundary conditions have new requirements due to the existence of additional air flow. The velocity inlet of the flow-follow device is set at the wall of the chamber to ensure uniform and stable airflow at the nozzle. The momentum value of the momentum source at the fan should be reduced because of the additional air flow at the nozzle to ensure that the speed at the nozzle can meet the requirements of pulsating working conditions. The flow velocity *vf* of the basic calculation example is 28 m/s, and the fan rotation speed is 168 r/min. A pressure outlet needs to be set on the wall of the chamber to meet the equilibrium state because the flow-follow device directly inputs fluid into the chamber. As shown in Fig. 38, a pressure outlet with an opening size of 4 m × 3 m is set on the rear wall of the parking chamber.

**Figure 39:**
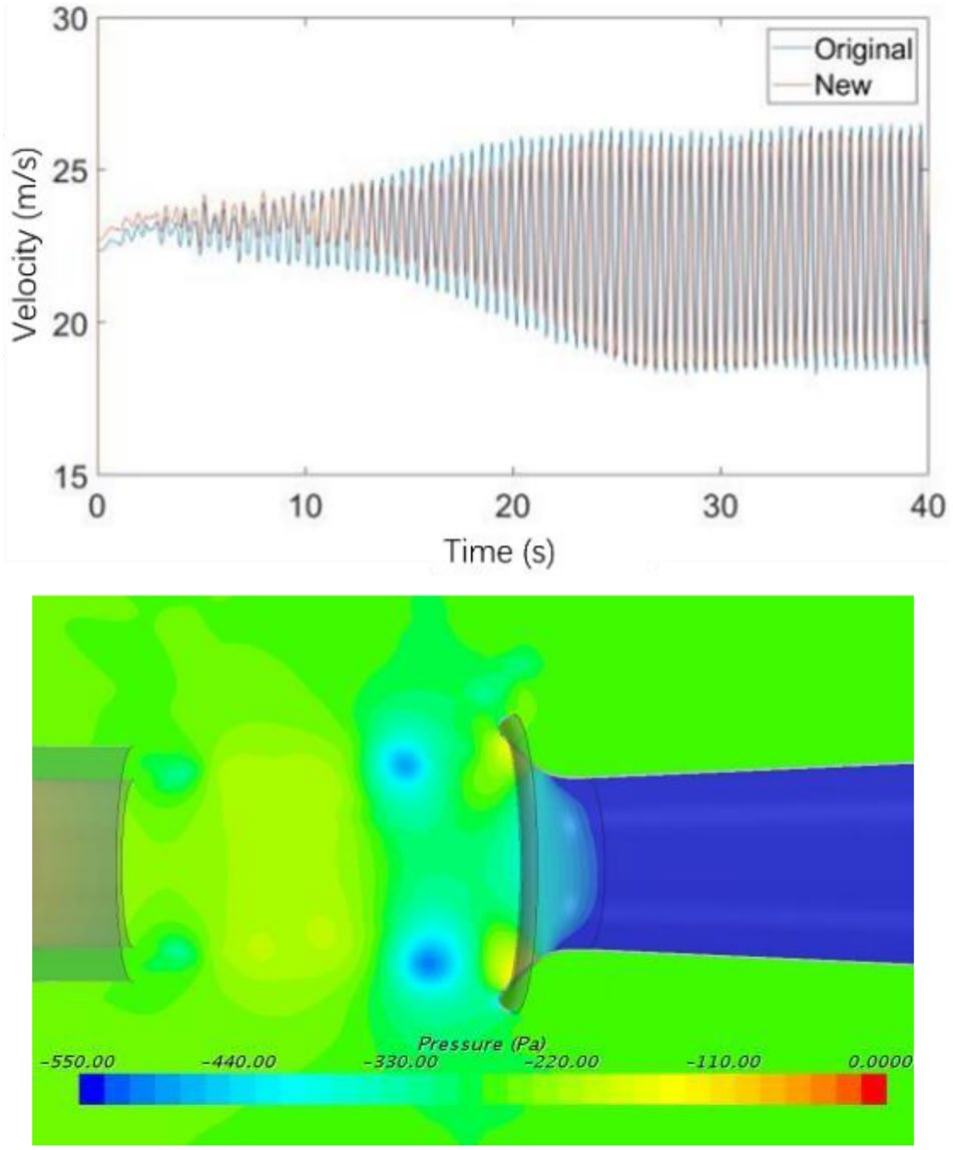
Simulation analysis of pressure outlet of flow-follow device.

The speed of the flow device is set to zero, and the pressure outlet is opened to simply explore whether the pressure outlet affects the recurrence of pressure pulsation and the flow field state. The results are shown in Fig. 39. The time domain velocity amplitude of the old and new models is the same, and pressure pulsation is still observed. Compared with the original simulation results, the flow field characteristics meet the expectations.

#### Results and discussion

**Figure 40:**
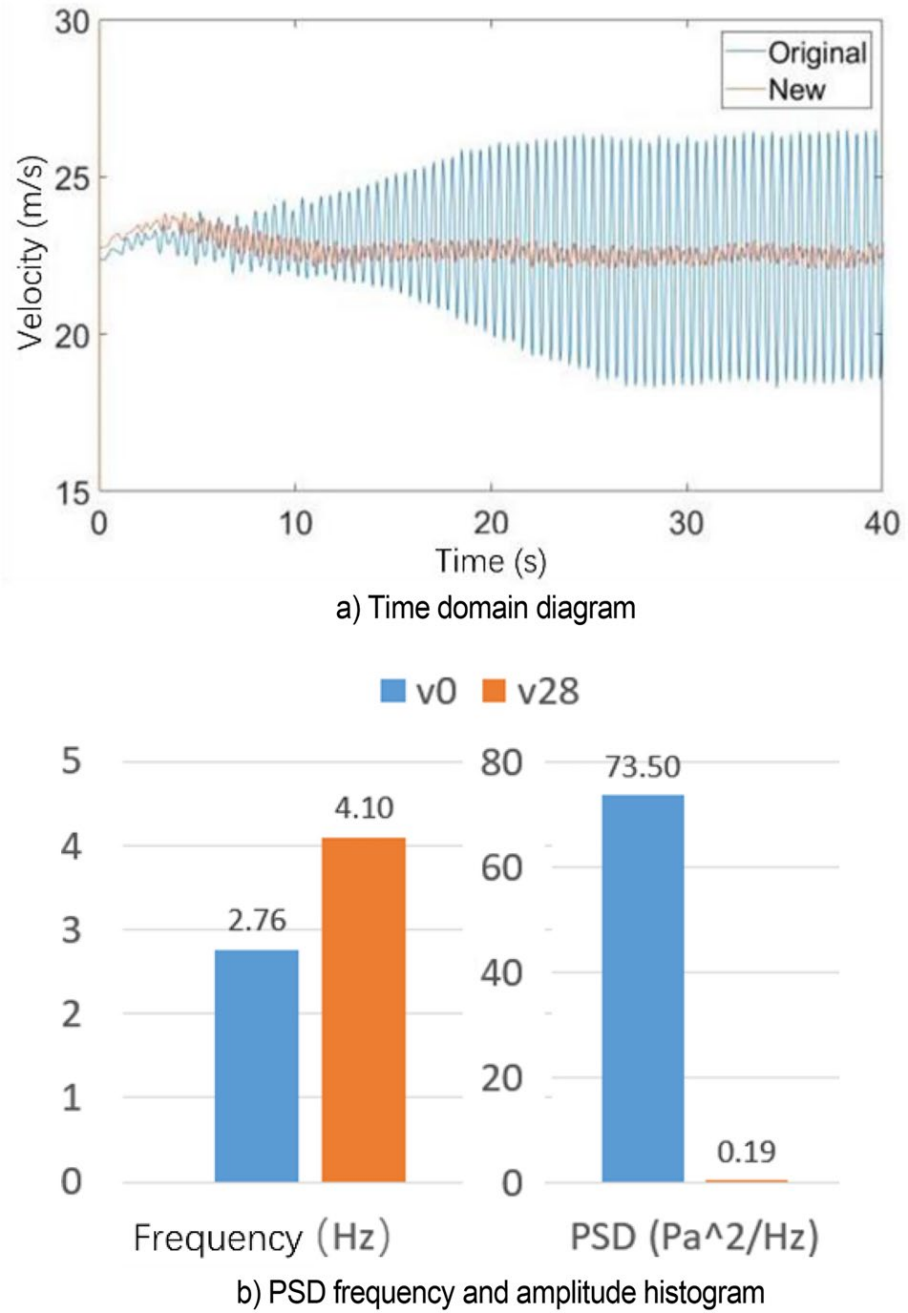
Simulation results of V28 working condition.

Figure 40a shows the velocity fluctuation diagram of v28 flow-follow device in the time domain. The velocity fluctuation at the nozzle is considerably reduced, greatly weakening the influence of low-frequency pressure fluctuation. As shown in Fig. 40, the main frequency of velocity pulsation is increased to 4.1 Hz by the application of flow-follow device, and no loop resonance is observed.

**Figure 41:**
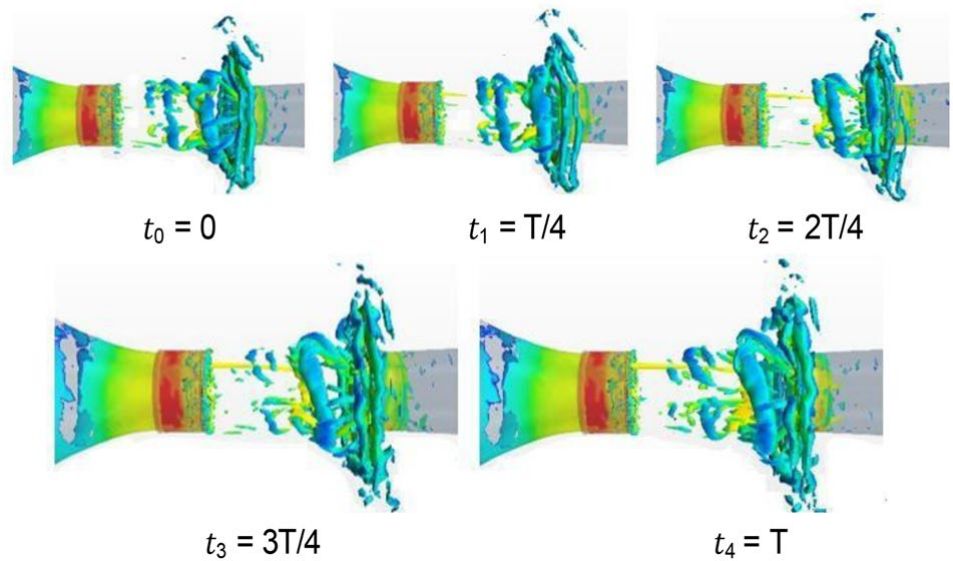
The isosurface cloud diagram of Q criterion with flow-follow device (Q = 200 /s^2^).

Figure 41 shows the Q-criterion isosurface cloud map of the flow device. As shown in Fig. 25, the modified device produces a double ring structure in the middle of the test section at time *t*1, but they are not complementary here. The vortex ring structure in the leading position is relatively complete and regarded as the main ring. The vortex ring structure in the backward position is relatively broken and is regarded as a secondary ring. In the process of further development of the flow field, the secondary ring is integrated into the main ring and smashed after hitting the collection port together.

**Figure 42:**
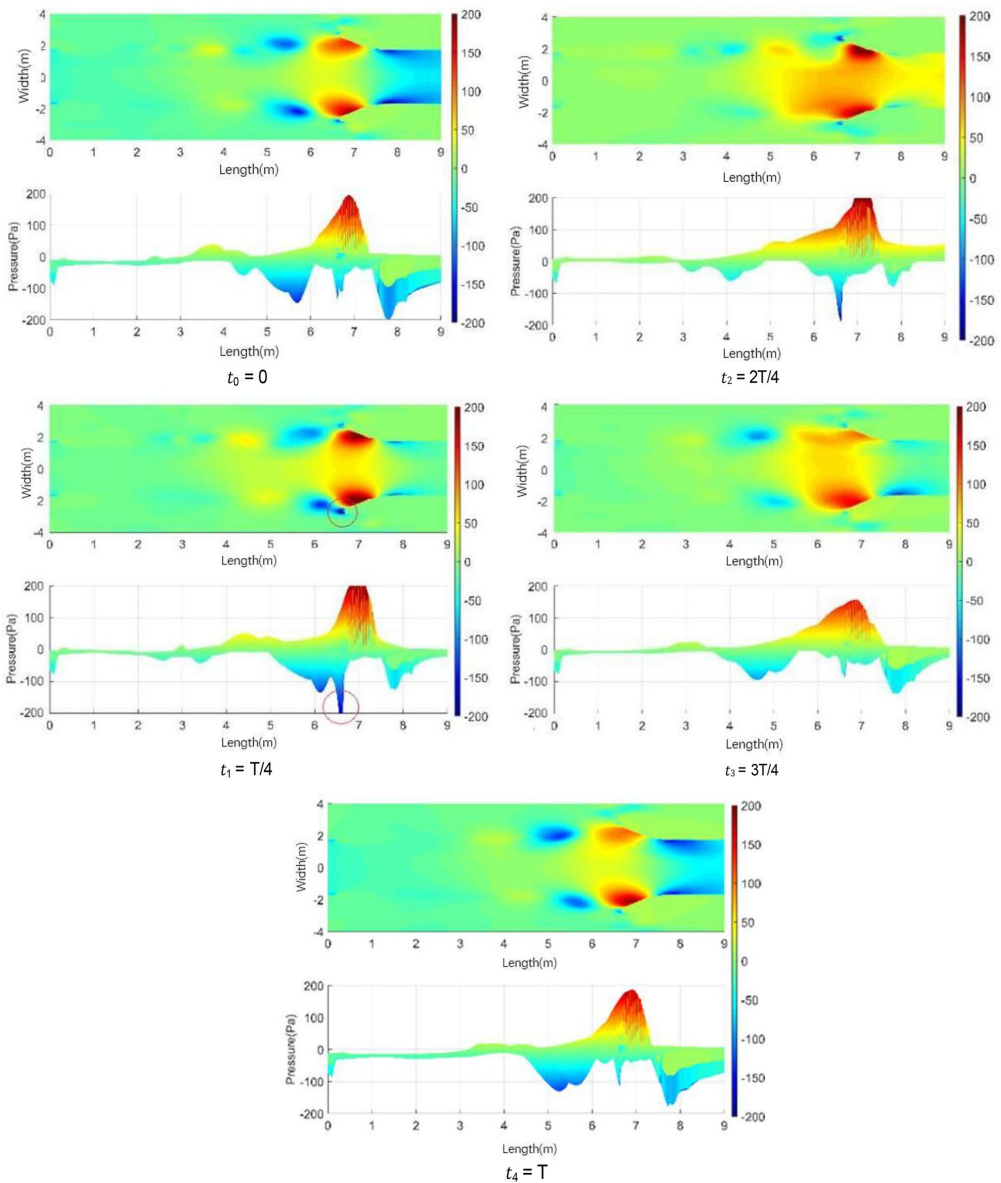
Pressure cloud diagram on the XY central plane of the nozzle flow-follow device.

**Figure 43:**
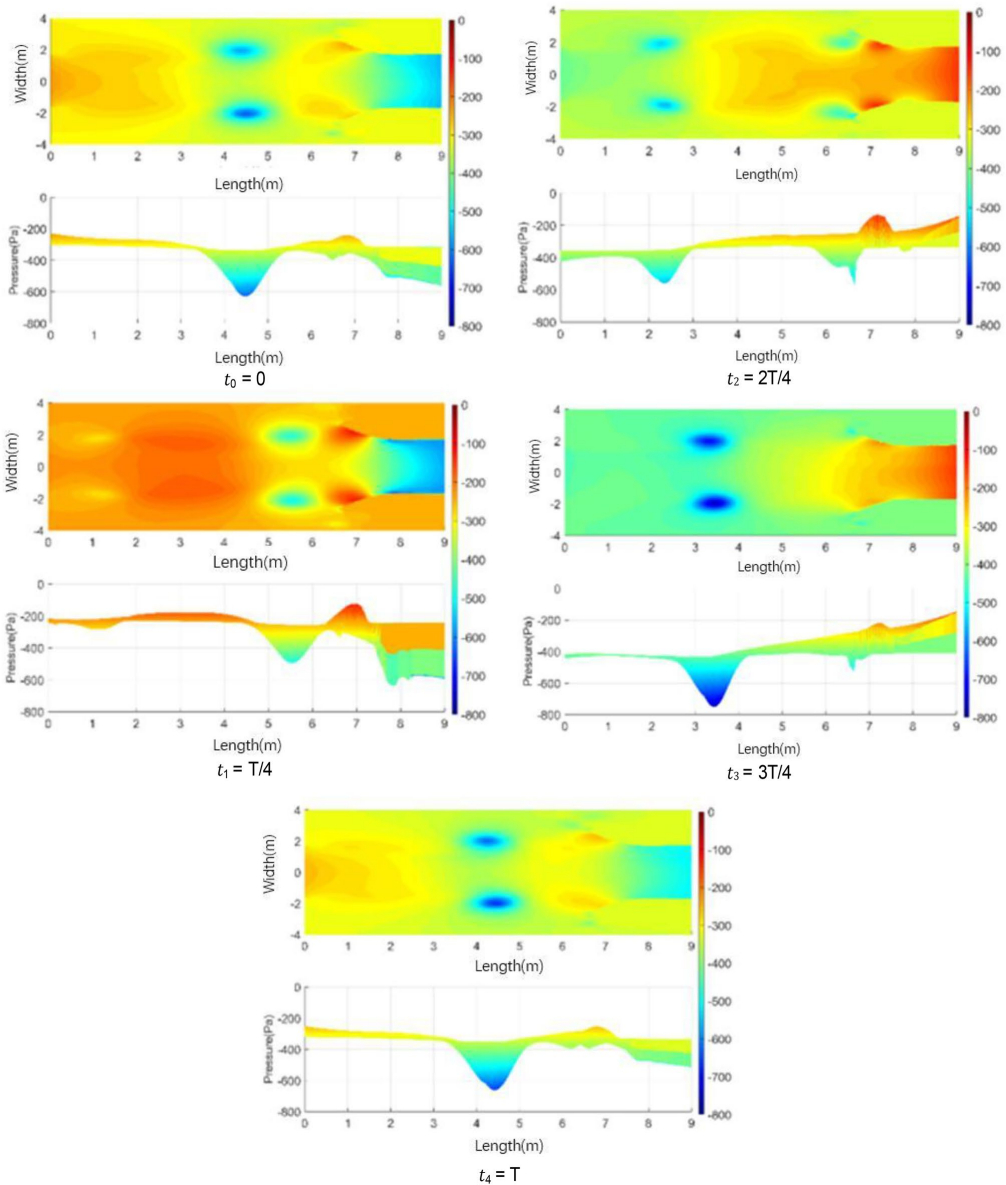
Pressure cloud diagram on the XY central plane.

Figure 42 shows the pressure cloud diagram on the XY central plane of the nozzle flow-follow device. In the whole pulsation cycle, the average pressure in the front section of the test section is maintained at about 0 Pa. In Fig. 43, it fluctuates in the range of − 400 and − 200 Pa. The maximum pressure extreme value in the pulsation is reduced from − 800 Pa to − 160 Pa, and the amplitude range of pressure fluctuation is greatly reduced. Under the support of the flow-follow device, the pressure pulsation has the extreme pressure (marked position at time *t*1) on the positive windward side of the collection port in the cycle, and this position still appears as the extreme pressure after the collection port is broken at time *t*3.

**Figure 44:**
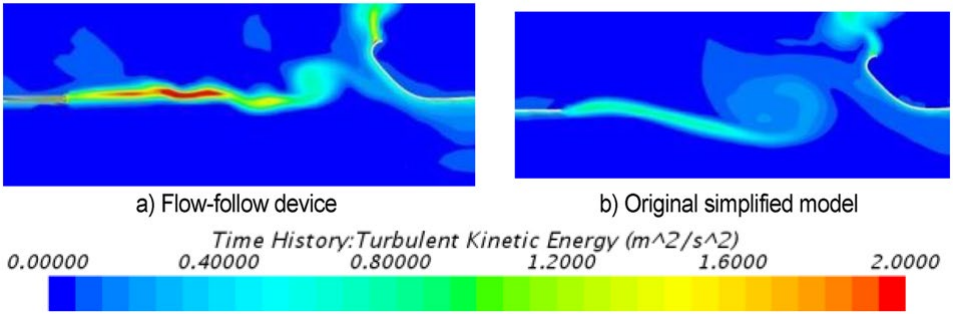
Cloud diagram of turbulent kinetic energy at the jet boundary.

Turbulent kinetic energy is an index used to measure the turbulent pulsation energy, which can be used to characterize the intensity and distribution of sound sources in the flow field. Figure 44 shows the cloud diagram of turbulent kinetic energy at the jet boundary with the flow-follow device and the simplified model. The jet position of the flow-follow device has a large turbulent kinetic energy value, indicating that new noise sources may be found at the jet boundary although the addition of the flow-follow device alleviates the pressure pulsation.

#### Analysis of influencing factors

**Figure 45:**
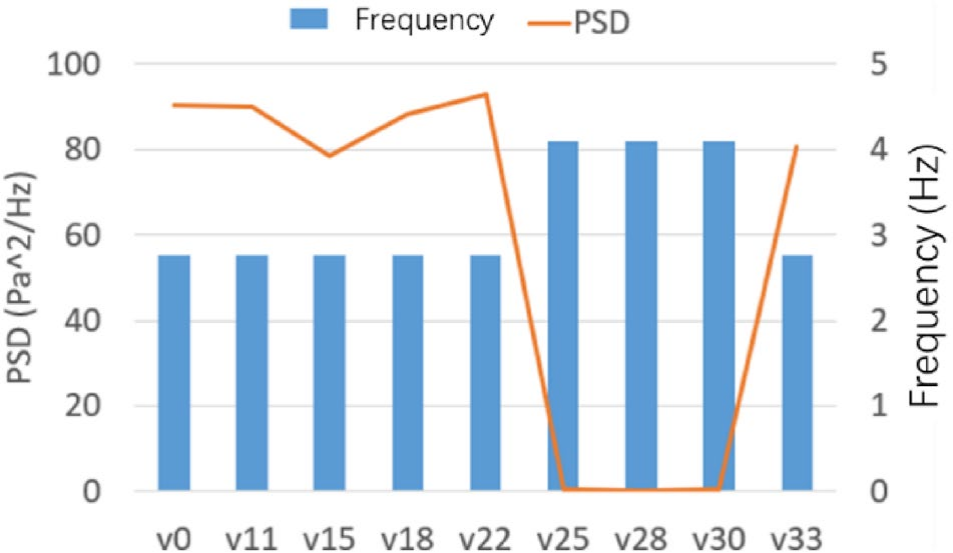
PSD histogram at each speed.

Figure 45 shows the power spectral density histogram at each speed. The velocity interval is 50%–150% of the nozzle speed of 22 m/s. When the flow speed is set to 25, 28, and 30 m/s, the pressure pulsation of the flow field is relieved, and the main frequency of pulsation is increased to about 4.1 Hz. Other working conditions do not affect the original jet state.

**Figure 46:**
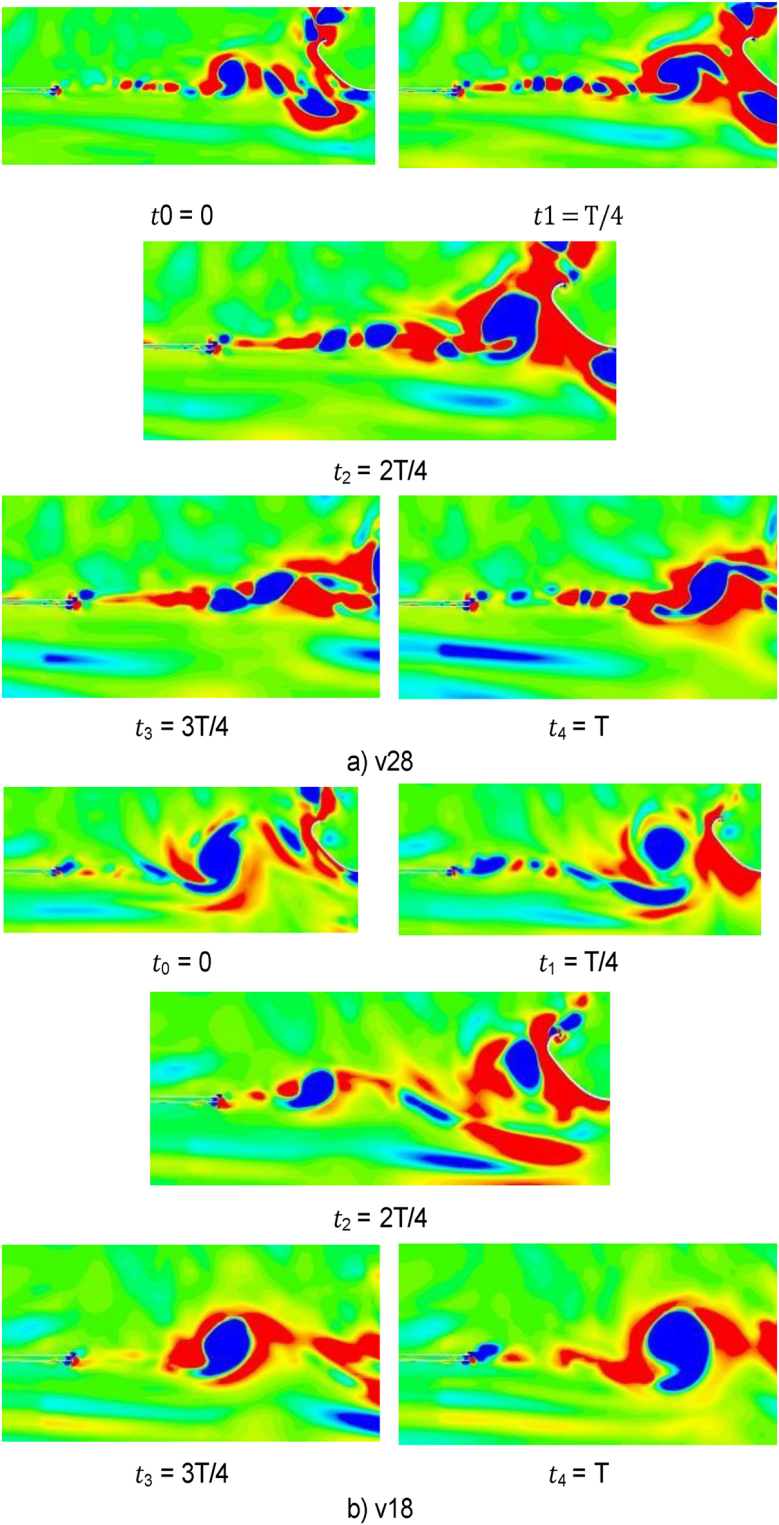
Single cycle lambda 2 cloud diagram of two representative working conditions.

Figure 46 shows the lambda 2 cloud diagram of two representative working conditions in single cycle. λ2(lambda 2) criterion is an improvement on the search pressure minimum area method. The Cauchy-Stokes method is the decomposition of velocity gradient tensor ∇V into symmetrical part A and antisymmetrical part B. A represents the deformation of the fluid and B represents the rotational motion of the fluid. λ2 criterion identifies vortices by judging whether there are two negative eigenvalues in combination equation A2 + B2 of symmetric and antisymmetric tensors. The eigenvalues are sorted according to λ1 > λ2 > λ3.When λ2 < 0, the pressure has a minimum value, that is, there is a vortex region. The v28 is beneficial to weaken the pressure pulsation. At *t*0, the tail vortex in the previous period induces a new jet at the nozzle after it collides with the collection port and breaks, and the vortex in the middle area of the test section continues to develop. At *t*1 and *t*2, the classical discontinuous jet vortex structure appears in the front and middle of the test section. The vortex in the tail section grows gradually and breaks after hitting the collection port at *t*3. At the same time, a new vortex is induced at the nozzle. The vortex in the middle section merges, grows, and enters the next cycle. The vortices produced by the jet in v18 fuse together previously.

**Figure 47:**
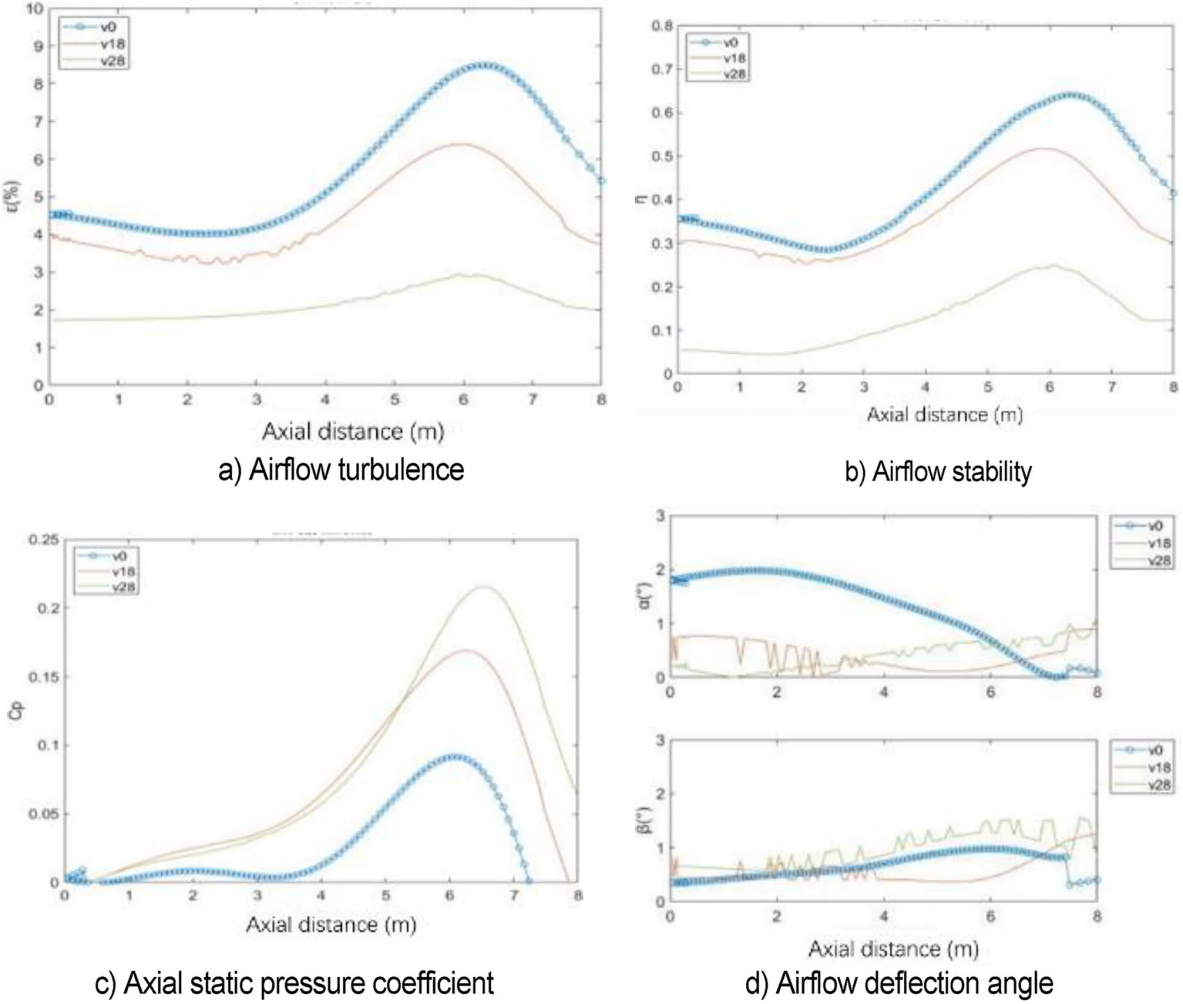
Flow field evaluation index.

Figure 47 shows the flow field quality coefficient diagram under v0, v18, and v28. As long as the flow-follow device is applied, the indexes of turbulence, stability, and deflection angle of the air flow are improved to a certain extent, and the effect of v28 is the best. However, the axial static pressure coefficient increases. The velocity distribution of the nozzle has a certain effect on the velocity uniformity of the core area due to the application of the flow-follow device.

## Conclusions

The pressure pulsation of open wind tunnel is a high-intensity vibration phenomenon in the low-frequency range. Although it is outside the common octave range, it still seriously affects the measurement accuracy of wind tunnel test and endangers the safety of equipment. Combined with the wind tunnel test data, this study uses CFD to explore the generation mechanism of low-frequency pressure pulsation and proposes a variety of vibration and noise reduction methods. The conclusions can be summarized as follows:

1. The phenomenon of low-frequency pressure pulsation has a certain velocity range and is caused by the edge feedback effect and pipeline resonance. The edge feedback effect is the most exciting source, and the pipeline circuit is the response. The edge feedback effect consists of the free jet motion at the nozzle and the pressure wave feedback at the collector.
2. In accordance with free jet theory, the spoiler at the nozzle can stagger the separation time of air flow at the edge of the nozzle. It destroys the integrity of the original vortex ring in the jet shear layer. A complementary double ring structure is formed. The trend of small vortex structure merging into large vortex structure can be delayed. The vibration mitigation effect is related to the length, number, and duty ratio of the device. Extremely large or extremely small number and duty ratio are unconducive to the damage of the vortex ring structure.
3. The flow-follow device at the nozzle can change the original jet boundary layer by compensating the energy loss. It reduces the jet diffusion angle and makes the air flow form the main and auxiliary ring structures in the middle of the test section. The strength of the main vortex ring is weaker than the original vortex ring structure. Its cushioning effect takes effect when the following speed is higher than the incoming speed.

## Data Availability

The datasets used and analysed during the current study available from the corresponding author on reasonable request.
